# Rejuvenating senescent hair follicles: a novel conjugated linoleic acid based nanovesicle approach to treat androgenic alopecia

**DOI:** 10.1186/s12951-025-03865-2

**Published:** 2025-11-26

**Authors:** Yating Dong, Yingying Sun, Aojie Li, Yuxuan Yu, Wengkuan U, Hongjuan Zhang, Xuefei Zhang, Yihua Huang, Haiyan Hu

**Affiliations:** 1 School of Pharmaceutical Sciences, Sun Yat-sen University, University, Town, Guangzhou, 510006 P. R. China; 2 School of Traditional Dai-Thai Medicine, West Yunnan University of Applied Sciences, Jinghong, 666100 P. R. China; 3 Guangdong Provincial Key Laboratory of Chiral Molecule and Drug Discovery, Sun Yat-sen University, University Town, Guangzhou, 510006 P. R. China; 4 State Key Laboratory of Anti-Infective Drug Discovery and Development, School of Pharmaceutical Sciences, Sun Yat-sen University, Guangzhou, 510006 P. R. China

**Keywords:** Androgenic alopecia, Conjugated linoleic acid, Anti-aging, Nanoplatform, Minoxidil

## Abstract

Existing therapies for androgenetic alopecia (AGA) fall short of expectations due to the complex pathogenesis, deficient drug enrichment, and obvious adverse effects. Although elevated dihydrotestosterone (DHT) is responsible for the miniaturization of hair follicles (HFs), cell aging has been found to be closely associated with AGA, however not received sufficient attention before. In this study, we first explored the deleterious effects of DHT on HFs, then identified the anti-aging potential of conjugated linoleic acid (CLA) from several polyunsaturated fatty acids. We then developed CLA-loaded nanovesicles (Arg-CLAVs) capable of counteracting DHT-induced HF aging. Arg-CLAVs rejuvenated DHT-induced senescent cells by alleviating oxidative stress, reducing the production of senescence-associated secretory phenotype, and modulating the MAPK-ERK signaling pathway. Moreover, Arg-CLAVs enhanced the HF niche by facilitating angiogenesis, preserving local oxidative homeostasis, and promoting the proliferation and migration of dermal papilla cells. Given its unique properties of nanocarriers, Arg-CLAVs exhibited better skin retention and HF targeting ability compared to the normal tincture. Subsequently, minoxidil (MNX) was loaded into the nanovesicle (MNX@Arg-CLAVs) and evaluated for its hair growth-promoting efficacy in vivo. Our results demonstrated that, in both male and female AGA mice, MNX@Arg-CLAVs with reduced MNX dosage and lower organic solvent contents, exhibited stronger effects on hair regeneration promotion and anti-aging in HFs, while inducing less formulation-associated skin irritation than commercial topical MNX tincture. In sum, this study developed a novel nanoplatform with HF anti-aging activity for AGA that enables synergistic effects with existing drugs.

**Graphical Abstract:**

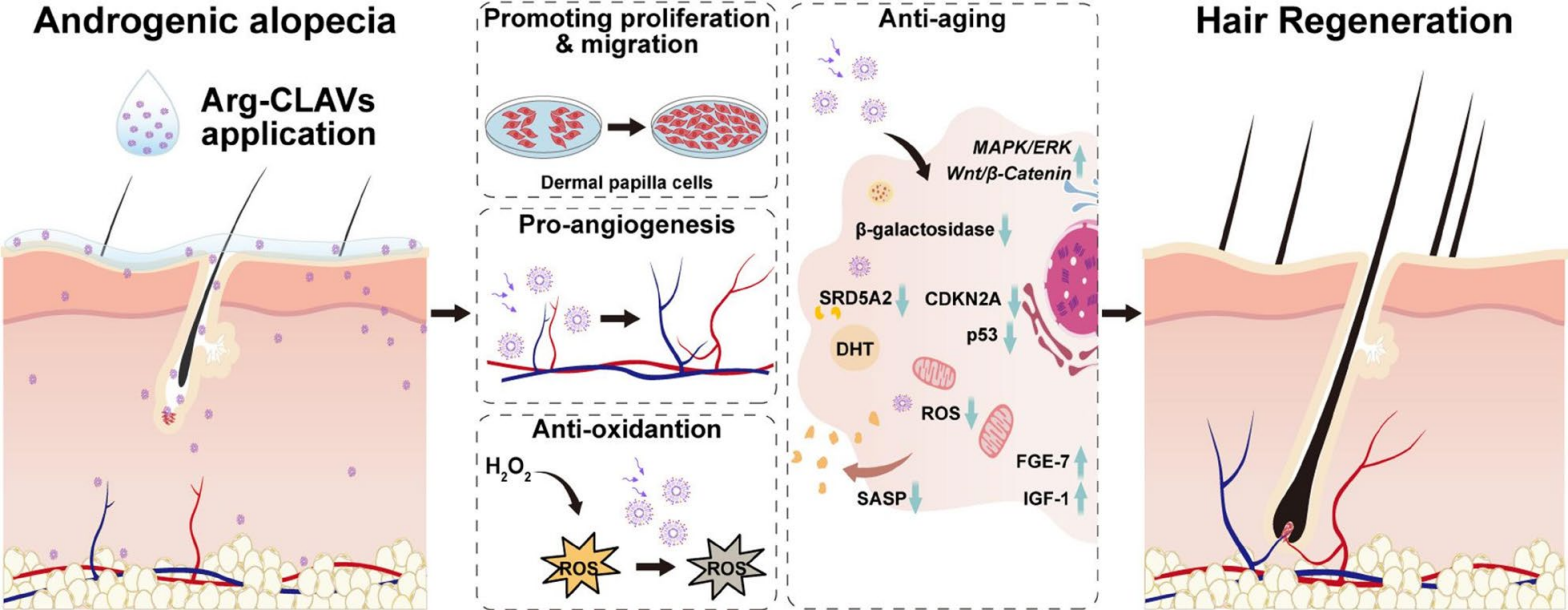

**Supplementary Information:**

The online version contains supplementary material available at 10.1186/s12951-025-03865-2.

## Introduction

Alopecia, a globally prevalent dermatological disorder affecting over 50% of the population, has been recognized to exert profound detrimental impacts on individuals’ mental health and overall quality of life. Notably, its incidence has exhibited a concerning upward trajectory, with increasing prevalence among younger people, a phenomenon potentially exacerbated by contemporary environmental stressors, including psychological strain and pollution exposure [1]. Androgenetic alopecia (AGA), constituting the predominant form of alopecia, is clinically characterized by progressive follicular miniaturization and shortened anagen duration. The etiology of AGA is linked to multifactorial pathophysiological mechanisms, with androgen dysregulation being identified as the primary driver. This aberrant hormonal status is principally mediated by the overexpression of type II 5α-reductase, an enzyme that catalyzes the conversion of testosterone (TS) to dihydrotestosterone (DHT) in scalp tissues. Compared to TS, DHT exhibits heightened binding affinity for the androgen receptor (AR), a property that facilitates pathological signaling [2]. The subsequent DHT-AR complexes have been shown to disrupt normal hair cycle regulation and induce perifollicular microvascular degeneration through paracrine signaling. Besides, androgens render hair follicles (HFs) more sensitive to oxidative stress, thereby accelerating their destruction. Therefore, we have developed a comprehensive anti-AGA strategy encompassing multiple aspects, including 5α-reductase inhibition, oxidative stress mitigation, cellular proliferation enhancement, and angiogenesis promotion to remodel the healthy HF niche and synergistically improve therapeutic outcomes in comparison to single-pathway approaches.

However, recent studies have identified cellular aging as a critical yet underestimated contributor to the pathogenesis of AGA. Senescent cells not only lose their regenerative capacity to initiate new hair growth cycles but also characteristically develop a senescence-associated secretory phenotype (SASP), thereby secreting various pro-inflammatory factors, disturbing the activation and differentiation of hair follicle stem cells (HFSC), ultimately resulting in irreversible progression of hair loss [3]. These findings indicate that HF aging constitutes a pivotal pathogenic mechanism in AGA etiology, yet this fact has not been given attention in clinical practice. Accordingly, the development of therapeutic approaches targeting senescence for AGA management attracts more concerted exploration.

Conjugated linoleic acid (CLA), a stereoisomeric mixture of linoleic acid and classified under *ω*−6 polyunsaturated fatty acids (PUFAs), has been designated by the FDA as a functional dietary supplement owing to its diverse array of therapeutic benefits, including anti-inflammatory activity, oxidative stress attenuation, blood pressure regulation, and cognitive enhancement [3–6]. Additionally, CLA was found to spontaneously assemble into a nanovesicular architecture, presenting great potential as a drug carrier [7].

Nanocarrier-based delivery systems have emerged as a focus in alopecia therapeutics, primarily attributed to their capacity to enhance transdermal permeation and reduce systemic toxicity through precise follicular targeting [8]. Notably, CLA is structurally analogous to follicular sebum lipids, which are hypothesized to enhance transdermal penetration by modulating lipid bilayer organization and increasing membrane fluidity [9, 10]. Additionally, most nanocarriers are often overly complex in design, which presents a significant challenge to their preparation process. In contrast, CLA-based nanovesicles can self-assemble *via* a simple ethanol injection method [11].

Minoxidil (MNX) is the only FDA-approved topical medication for AGA treatment, primarily functioning through mechanisms such as promoting cell proliferation, angiogenesis induction, and activation of the Wnt/β-catenin signaling to suppress alopecia progression [12]. Despite nearly four decades of clinical use, the efficacy of topical MNX remains suboptimal, with highly variable responses in promoting hair regrowth [13]. Moreover, there is insufficient evidence supporting the efficacy of MNX in alleviating DHT-mediated follicular damage and cellular aging, implying that even with MNX treatment, DHT continues to inflict progressive damage on follicular structures and induce follicular cell senescence. Given these issues, we speculated that Arg-CLAVs could serve not only as a promising nanovesicle for inhibiting HF aging, but also as a platform to address the shortcomings of existing treatments.

Accordingly, this study develops a multifunctional nanovesicle (Arg-CLAVs) by the co-assembly of CLA, phospholipids, and polyethylene glycol succinate (TPGS), with arginine (Arg) absorbed on the outer layer (Scheme 1). The nanovesicles achieve enhanced drug deposition at HFs *via* sebum-lipid interaction and the nanoparticle ratchet effect. Subsequently, positively charged arginine coating facilitates the rapid endocytosis of Arg-CLAVs, thereby improving the delivery efficiency of encapsulated active ingredients. Functionally, Arg-CLAVs promote angiogenesis, scavenge ROS, and enhance DPC proliferation and migration—collectively remodeling a favorable niche for HFs regrowth. By downregulating the hair-growing negative gene expression and suppressing the SASP levels in DPCs, the nanovesicles mitigated DHT-induced pathological senescence in DPCs. Furthermore, MNX was encapsulated into Arg-CLAVs to construct MNX-loaded nanovesicles (MNX@Arg-CLAVs). Notably, the Arg-CLAVs platform overcame critical limitations of conventional MNX therapy against HFs aging. By integrating anti-aging with remodeling the HF niche, this nanovesicle represents a promising and innovative strategy for achieving comprehensive, long-lasting therapeutic outcomes in AGA management with enhanced biosafety.

**Fig. 1:**
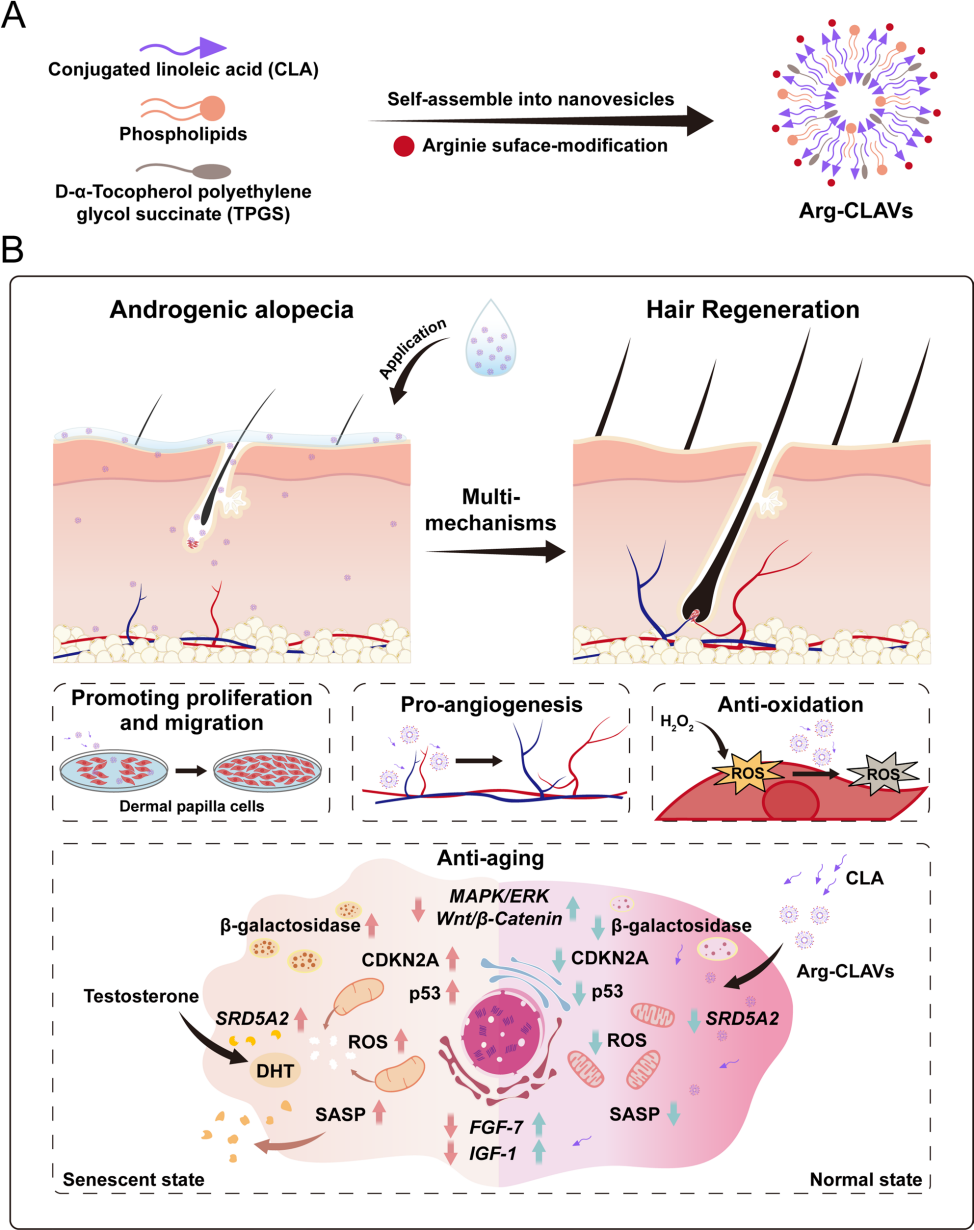
The schematic illustration of Arg-CLAVs, including its construction (**A**), and action mechanism on AGA treatment (**B**)

## Materials and methods

### Materials

Linoleic acid (LA), conjugated linoleic acid (CLA), α-Linolenic acid (ALA), minoxidil (MNX), vitamin E polyethylene glycol succinate (TPGS), trifluoroacetic acid, and 4,6-bis (imino)−2-phenylindole (DAPI) were purchased from Macklin (Shanghai, China). Arginine (Arg), coumarin 6 (C6), 2, 2’-azino-bis (3-ethylbenzothiazoline-6-sulfonic acid) (ABTS), dihydrotestosterone (DHT), and testosterone were obtained from Aladdin (Shanghai, China). Docosahexaenoic acid (DHA) and eicosapentaenoic acid (EPA) were purchased from Bidepharm (Shanghai, China). Ascorbic acid, β-galactosidase staining kit, and Hoechst 33342 were obtained from Beyotime (Shanghai, China). Egg yolk lecithin (EPC) was purchased from A.V.T. (Shanghai, China). Isoflurane was obtained from RWD Life Science (Shenzhen, China). Phosphotungstic acid was purchased from Shanghai Yuanye Bio-Technology (Shanghai, China). Minoxidil tincture (Mandi^®^, 5%) was obtained from Zhejiang Sunshine Mandi Pharmaceutical (Zhejiang, China). Diphenyl picrylhydrazine (DPPH) was purchased from TCI (Japan). CK19 antibody (YM3051) and *p53* (YT3528) antibody were obtained from Immunoway (USA). Ki67 antibody (261202) was purchased from ZenBio (Chengdu, China). ELISA assay kits were obtained from ELK Biotechnology (Wuhan, China). Trizol was purchased from Accurate Biotechnology (Hunan, China). Reverse transcription kit and SYBR Green PCR kit were obtained from MIKX (Shenzhen, China).

### Cell culture and animals

Human dermal papilla cells (HDPCs) were cultured in DEME medium (Gibco, USA) supplemented with 10% fetal bovine serum (FBS, PAN, South America), 100 U/mL penicillin, and 100 µg/mL streptomycin (Hyclone, USA) at 37 °C with 5% CO_2_. Human umbilical vein endothelial cells (HUVECs) were cultured in Endothelial Cell Medium (ECM) (ScienCell, USA) containing 5% fetal bovine serum (FBS, PAN, South America), 100 U/mL penicillin, and 100 µg/mL streptomycin (Hyclone, USA) at 37 °C with 5% CO_2_.

All animals were provided by the Laboratory Animal Center of Sun Yat-sen University (Guangzhou, China). All the animal procedures were conducted according to the laboratory animal regulations approved by the Institutional Animal Care and Use Committee of Sun Yat-sen University (Ethics Approval No. SYSU-IACUC-2023-001623).

### Screening of PUFAs

The activity of polyunsaturated fatty acids (PUFAs) in inhibiting DHT-induced cell damage and promoting the proliferation of HDPCs was evaluated using the CCK8 assay. Briefly, HDPCs were seeded in 96-well plates at a density of 5.0 × 10³ cells per well. The attached cells were treated with culture medium containing both DHT and the tested drugs. After 24 incubation, cell viability was assessed using the CCK8 (No. K1018, APExBIO, USA) assay. For the proliferation test, the HDPCs were seeded into a 96-well plate at a density of 3.0 × 10³ cells per well and cultured for 24 h. Subsequently, various PUFAs solutions were added to the cells, while the blank control group was supplemented with an equal volume of culture medium. A blank well without cells was set as the zeroing group. After administration, the samples were cultured in an incubator for 24 h. Then, the culture medium was discarded, and 200 µL of DMEM medium containing 1% CCK-8 (v/v) was added to each well. After incubation at 37 °C in the dark for 2 h, the absorbance at 450 nm was measured using an enzyme detector (Synergy H1, Bio Tek, USA), and the cell viability was calculated according to the following formula 1.

1 $$\text{Cell viability}\, (\%)=\frac{{\text{OD}}_{\text{Exp}}\text{-}{\text{OD}}_{\text{ZC}}}{{\text{OD}}_{\text{NC}}\text{-}{\text{OD}}_{\text{ZC}}}$$

where *OD*_*Exp*_ represents the absorbance value of each experimental group, *OD*_*ZC*_ is the absorbance value of the zeroing group, and *OD*_*NC*_ is the absorbance value of the blank control group.

### Preparation of CLA-based nanovesicles

Arg-CLAVs were fabricated through the solvent injection method. To put it simply, CLA (5 mg/mL), TPGS (1.25 mg/mL), and EPC (1.25 mg/mL) were dissolved in anhydrous ethanol. Subsequently, with continuous stirring, the mixed solution was added dropwise into the phosphate buffered saline (PBS) buffer (pH 6.5). After further stirring for 30 min, CLAVs were obtained. For the preparation of Arg-CLVAs, the ethanol mixture solution was dropped into PBS buffer containing Arg (5 mg/mL). When preparing MNX@Arg-CLAVs, MNX was added to the mixture of ethanol solution.

### Characterizations of CLA-based nanovesicles

The hydrodynamic diameter and zeta potential of CLAVs, Arg-CLAVs, and MNX@Arg-CLAVs were measured by the dynamic light scattering (DLS) method using ZetaSizer Nano Series 90 (Malvern Instrument, Malvern, UK), while the morphological characteristics of MNX@Arg-CLAVs were observed by transmission electron microscope (TEM) (HT7800, HITACHI, Japan) after dyeing with phosphotungstic acid. To investigate the existence form of Arg in Arg-CLAVs, samples were freeze-dried into a solid powder, subsequently scanned by Fourier Transform Infrared Spectroscopy (FTIR) (NICOLET 6700, Thermo Fisher Scientific, USA). To determine the encapsulation efficiency (EE%) and drug loading (DL%), samples were demulsified with methanol, and then the drug contents were analyzed by high performance liquid chromatography (HPLC, Agilent 1260, Agilent Technologies, USA). When evaluating the stability of nanovesicles, samples were stored at room temperature and 4 °C, and the particle sizes, PDI, and zeta potential were recorded over 90 days.

### In vitro transdermal study

Porcine skin and rat skin have been recognized as valid substitutes for the prediction of human skin permeability because of their physiological and histological similarities to human skin [14], wherein porcine skin has the highest similarity [15] in pharmacological release. Here, we conducted a transdermal visualization experiment on rat abdomen skin, and carried out the transdermal quantitative analysis on Bama pig abdomen skin. The Bama pig abdomen skin was frozen and stored in our laboratory. To characterize the skin permeation of Arg-CLAVs, the excised rat abdominal skin was fixed on the Franz diffusion cell with an effective diffusion area of 3.14 cm², and 8 mL of normal saline containing 40% PEG-400 (v/v) was used as the receiving medium. The system was equilibrated at 32 °C and 250 rpm for 30 min. C6 was loaded into Arg-CLAVs in place of MNX and was added to donor cells. The skin was taken out at 4 h, 8 h, and 12 h, and cryosections were prepared for observation.

Additionally, using the same method, 500 µL of MNX@Arg-CLAVs or MNX tincture was added to the donor compartment. Samples were collected at 0 h, 4 h, 8 h, 12 h, and 24 h, followed by the addition of an equal volume of deionized water. At specific time points, the receiving medium was collected. The stratum corneum of the cleaned skin was stripped with adhesive tape, and then transparent tape was used to press cyanoacrylate adhesive into the pig skin follicles. After the adhesive dried completely, the tape was removed, soaked with methanol, and ultrasonicated to extract MNX to investigate the retention of the drug in the stratum corneum and HFs. Finally, the remaining pig skin was cut into pieces, and MNX in the skin was extracted with methanol by ultrasonication to investigate the retention of the drug in the remaining skin tissue. The content of MNX in each part was detected by HPLC, and the follicular targeting factors were calculated according to formula 2:

2 $$\text{Follicular targeting factors}\frac{{{m}}_{{HF}}}{{{m}}_{{HF}}\text{+}{{m}}_{{SC}}{+}{{m}}_{{RS}}}$$

where *m*_*HF*_ represents the content of MNX at the HFs, *m*_*SC*_ represents the content of MNX in the stratum corneum, and *m*_*RS*_ represents the content of MNX in the remaining skin tissue.

### Cell proliferation study

The CCK8 assay was used to assess the impact on the proliferation of HDPCs and HUVECs. HDPCs and HUVECs were seeded in 96-well plates at densities of 3.0 × 10³ cells per well and 5.0 × 10³ cells per well, respectively. After overnight adherence, different concentrations of MNX, CLA, and Arg-CLAVs solutions were added to each well. The DMEM complete medium without any drugs was used as the negative control group, and MNX (with a final concentration of 20 µg/mL) was used as the positive control group, meanwhile the cell-free blank wells were set as the zeroing group. Finally, cell viability was assessed using the CCK8 assay at 24 h. Each group is set up with 6 parallel trials, and this experiment should be repeated at least 3 times.

### Cell migration experiment

The effect of nanovesicles on cellular migration was assessed *via* scratch wound assay. HDPCs and HUVECs were seeded in 6-well plates at densities of 5.0 × 10⁴ cells per well and 1.0 × 10⁵ cells per well, respectively. When the cells were attached and covered the bottom of the wells, three straight lines were drawn in the middle of each well, and then the cells were washed with PBS. Different concentrations of Arg, CLA, CLAVs, and Arg-CLAVs solutions were added to each well, with the final concentration of MNX being 20 µg/mL. The drug was diluted to the desired concentration using a low-serum DMEM medium containing 4% FBS. The medium without the drug served as the negative control group, while the MNX group was the positive control group. After incubation, wound photographs were taken at specific time points, and the migration distance was quantified using ImageJ. Each group is set up with 3 parallel trials, and this experiment should be repeated at least 3 times.

### HUVECs tubule formation experiment

Matrigel and DMEM medium were mixed at a volume ratio of 1: 1 and seeded at a volume of 50 µL per well at the bottom of a 96-well plate. The plate was then incubated in an incubator for more than 1 h to solidify. Next, HUVECs suspensions containing 1.5 × 10^5^ cells mixed with MNX, Arg, CLA, CLAVs, and Arg-CLAVs were seeded into each well, with the final concentration of MNX being 50 µg/mL. The control group was treated with a complete ECM without any drugs. After incubation for 4 h, the formation of blood vessels was observed under a microscope. The number of nodes, junctions, and total branch length of tubes wasquantified using ImageJ. Each group is set up with 3 parallel trials, and this experiment should be repeated at least 3 times.

### Cell uptake study

Cell uptake was quantified by flow cytometry. Briefly, HDPCs and HUVECs were seeded and cultured overnight. Subsequently, the cells were treated with complete ECM containing C6@CLAVs (CLAVs encapsulating coumarin 6) or C6@Arg-CLAVs (with a final concentration of C6 at 1 µg/mL) for 4 h. After incubation, the cells were harvested, and the fluorescence intensity was measured using a confocal microscope (CLSM, Olympus, Japan).

### Scavenging effects on free radicals

According to the experimental protocol proposed previously [16], we first evaluated the in vitro antioxidant activity of nanovesicles by their levels of scavenging free radicals. In brief, CLA, TPGS, Arg, CLAVs, and Arg-CLAVs were diluted to the required concentrations, with anhydrous ethanol as the negative control, and ascorbic acid at a final concentration of 500 µg/mL as the positive control. Equal volumes of DPPH solution or ABTS^+^ working solution were added, and the anhydrous ethanol solutions of each component without DPPH or ABTS^+^ were set as the zero-adjustment groups. After incubation in a shaking incubator at 37 °C for 3 h, the absorbance was measured at 517 (DPPH) or 734 (ABTS^+^) nm using a microplate reader, and the free-radical scavenging rate of each component was calculated according to formula 3:

3 $$\text{Free radical scavenging}\,(\%)=\frac{{{OD}}_{{Exp}}{-}{{OD}}_{{ZC}}}{{{OD}}_{{NC}}{-}{{OD}}_{{ZC}}}$$

where *OD*_*Exp*_ represents the absorbance value of each experimental group, *OD*_*ZC*_ is the absorbance value of the zero-adjustment group, and *OD*_*NC*_ is the absorbance value of the negative control group.

### Scavenging effects on H_2_O_2_-induced ROS

The ROS levels in injured cells were detected by the fluorescence staining method. Briefly, HDPCs were cultured in glass dishes at a density of 1 × 10^5^ cells/well. After attachment, the cells were incubated with Arg, CLA, CLAVs, and Arg-CLAVs (with a final concentration of 200 µg/mL for Arg-CLAVs) and exposed to hydrogen peroxide (H_2_O_2_). After 24 h of incubation, DCFH-DA probe (10 µM, D6470, Solarbio, China) was added, and the cells were further incubated for 30 min. Followingly, DAPI (5 µg/mL) was used for nuclear staining. Subsequently, the cells were observed under CLSM [Ex = 488 nm (DCFH-DA), Ex = 405 nm (DAPI)].

### Anti-cell damage activity

The cell viability was determined by the CCK8 assay to assess whether nanovesicles protect cells from DHT-induced damage. Briefly, HDPCs were seeded in 96-well plates at a density of 5.0 × 10³ cells per well. The attached cells were treated with culture medium containing both DHT and the tested drugs. After 24 h-incubation, cell viability was assessed using the CCK8 assay.

### Anti-cell senescence activity

The cellular senescence status was detected using a β-galactosidase staining kit (Beyotime, C0602, China). Briefly, HDPCs were seeded into the bottom of a 12-well plate at a density of 5.0 × 10⁴ cells per well. After attachment, the cells were treated with blank medium (without serum and antibiotics) containing different drugs. After 24 h of incubation, the medium was replaced with both DHT and the drugs diluted in blank medium. After another 48 h-incubation, the cells were stained using the β-galactosidase staining kit, captured under a microscope, and the positive rate of β-galactosidase was calculated using ImageJ.

### Alleviation of DHT-induced oxidative stress

The cellular oxidative stress level was evaluated using Hoechst 33342 staining. Briefly, HDPCs were seeded into the bottom of confocal dishes at a density of 1.0 × 10⁵ cells per well and incubated with DHT solution for 4 h. The medium was then replaced with a medium containing both DHT and the drugs. After another 24 h of incubation, the cells were stained with DCFH-DA probe (10 µM) for 30 min. After washing away the excess stain, the cells were stained with Hoechst 33342 (10 µg/mL) for 10 min. Finally, the cells were observed under CLSM.

### Western blotting analysis

The protein expression levels of p-ERK1/2 and ERK1/2 were evaluated through Western blotting. HDPCs were seeded in 6-well plates at densities of 2 × 10^5^ cells per well. Once the cells adhered and covered the well bottom, the cells were treated with blank culture medium, DHT, CLA, and Arg-CLAVs. For the CLA and Arg-CLAVs groups, DHT was added first to establish the model, followed by the addition of the respective drugs. Subsequently, protein samples extracted from HDPCs as determined by BCA assay, was lysed using RIPA buffer. The protein samples were then separated *via* SDS-PAGE gels and transferred onto nitrocellulose membranes. The membranes were incubated with primary antibodies: anti-p-ERK1/2 and anti-ERK1/2 overnight at 4 °C. After washing the membranes three times with TBST, the membranes were probed with their respective secondary antibodies. The anti-rat IgG HRP-linked antibody was incubated with the membranes at room temperature, followed by washing with TBST. The labeled proteins were visualized using a Bio-Rad imaging system (Servicebio, W2000, China). Each experiment was performed in triplicate.

### Impact on DHT-induced SASP levels

The levels of senescence-associated secretory phenotype (SASP) produced by DHT-induced HDPCs were detected using an ELISA kit, which included interleukin-6 (IL-6), tumor necrosis factor-alpha (TNF-α), and transforming growth factor-beta 1 (TGF-β1). Briefly, HDPCs were seeded into the bottom of a 96-well plate at a density of 1.0 × 10⁴ cells per well. Then, cells were cultured in medium containing both DHT and the drug for another 24 h, and the SASP contents were quantified according to the ELISA kit instructions.

### Impact on the expression of hair growth-related genes

The expression of DHT-induced related genes [*SRD5A2*,* β-Catenin*, Fibroblast growth factor-7 (*FGF-7)*, Insulin-like Growth Factor 1 (*IGF-1)*] and cell cycle related genes (*CDKN2A* and *p53*) in HDPCs wereexamined using real-time fluorescence quantitative PCR (RT-qPCR). Briefly, HDPCs were seeded into the bottom of a 6-well plate at a density of 1.0 × 10⁵ cells per well. Then, the medium containing both DHT and the drug was added. Subsequently, cellular total mRNA was extracted using the Trizol method, and then reverse transcribed into cDNA using a reverse transcription kit and a SYBR Green PCR kit. The data were normalized to the level of GAPDH. The primers were synthesized by Beijing Tsingke Biotechnology (China), of which the sequences employed for this investigation could be found in Table S1.

### RNA extraction and RNA sequencing analysis

Total RNA was extracted from the excised mouse dorsal skin using the Trizol kit. Sequencing libraries were generated using the TruSeq RNA Sample Preparation kit (Illumina) and then sequenced with the NovaSeq 6000 sequencer (Illumina). The Fastp software removes the adapter sequences and low-quality sequences from the raw data obtained from the sequencing, resulting in CleanData. R-packages-factoextra was used to perform principal component analysis (PCA). DEseq2 was used to conduct a significant difference analysis between the samples. Genes with a fold change (log_2_FoldChange) > 1 and *P* < 0.05 are defined as differentially expressed genes, and then are analyzed by Gene Ontology (GO) and Kyoto Encyclopedia of Genes and Genomes (KEGG) enrichment. Gene Set Enrichment Analysis (GSEA) was used for gene enrichment analysis.

### Construction of the AGA mice model

As previously reported, 7-week-old male C57BL/6J mice were randomly divided into 6 groups (*n* = 5). Following anesthesia, the dorsal hair of mice (approximately 2.0 cm × 4.0 cm) was shaved using an electric hair clipper, and depilatory cream was applied for complete hair removal. One day after recovery, each mouse was topically applied with 200 µL of saline or testosterone solution (TS, 5 mg/mL). After 30 min, the mice in the control and model groups were topically smeared with another 200 µL of saline, and the other mice were treated with 200 µL of different formulations, including 5% MNX tincture (commercial MNX tincture, containing 50 mg/mL of MNX), 0.45% MNX tincture (containing 4.5 mg/mL of MNX), Arg-CLAVs (containing 5 mg/mL of Arg-CLAVs), and MNX@Arg-CLAVs (containing 5 mg/mL of Arg-CLAVs with 4.5 mg/mL of MNX).

The female AGA mice model was established as described above. Briefly, 7-week-old female C57BL/6J mice were randomly divided into 4 groups (*n* = 4). Following the shaving procedure, each mouse received a topical application of 200 µL of normal saline or TS (5 mg/mL). After a 30-min interval, another 200 µL of different formulations was topically applied to the mice, which included normal saline, 5% MNX tincture (containing 50 mg/mL of MNX), and MNX@Arg-CLAVs (containing 5 mg/mL of Arg-CLAVs and 4.5 mg/mL of MNX).

### Assessment of hair regrowth

C57BL/6J mice exhibit skin pigmentation changes closely associated with the hair cycle, where the transition of HFs from telogen to anagen phase induces progressive darkening of the dorsal skin from pink to deep gray [17]. Therefore, the hair cycle progression was evaluated using a standardized skin color scoring method.

After the model was established and treatment was initiated, the hair regrowth in the depilated areas on the backs of male and female mice was observed at specific time points. On days 13 and 17, the skin color scores in the depilated areas of the mice were evaluated. On day 27 after treatment in male mice and day 34 after treatment in female mice, the mice were anesthetized, and the regrown hairs in the depilated areas were collected five times using adhesive tape. Subsequently, the regrown hairs were shaved off with an electric clipper, and their weight was measured.

### Histological evaluation

When the treatment cycle ended, the animals were sacrificed by cervical dislocation. The skin from the depilated dorsal region was collected, wrapped in aluminum foil, and fixed in 4% paraformaldehyde. The paraffin-embedded tissues were sectioned and subsequently stained with hematoxylin-eosin (H&E) and Masson’s trichrome staining. The expression of Ki67 in the skin tissues was characterized using immunofluorescence staining, while the expression of CK19 and *p53* was characterized using immunohistochemical staining. The sections were examined using a cellular imaging system.

### In vivo biosafety assessment

The in vivo safety evaluation was carried out along with the male animal experiments. The body weights of mice were recorded following each modeling and drug administration procedure. At the endpoint of the experiment, the mice were humanely euthanized, and their main organs (heart, liver, spleen, lung, and kidney) were harvested and fixed in 4% paraformaldehyde. The tissues were embedded in paraffin, sectioned, and stained with H&E. The sections were observed using a cell imaging system, and relevant images were taken.

### Statistical analysis

All data were expressed as means ± standard deviations (SD). Significant differences were assessed by one-way analysis of variance (ANOVA), followed by Tukey’s multiple comparison test using GraphPad Prism version 9 software.

## Results

### DHT induced cell senescence in DPCs

**Fig. 2:**
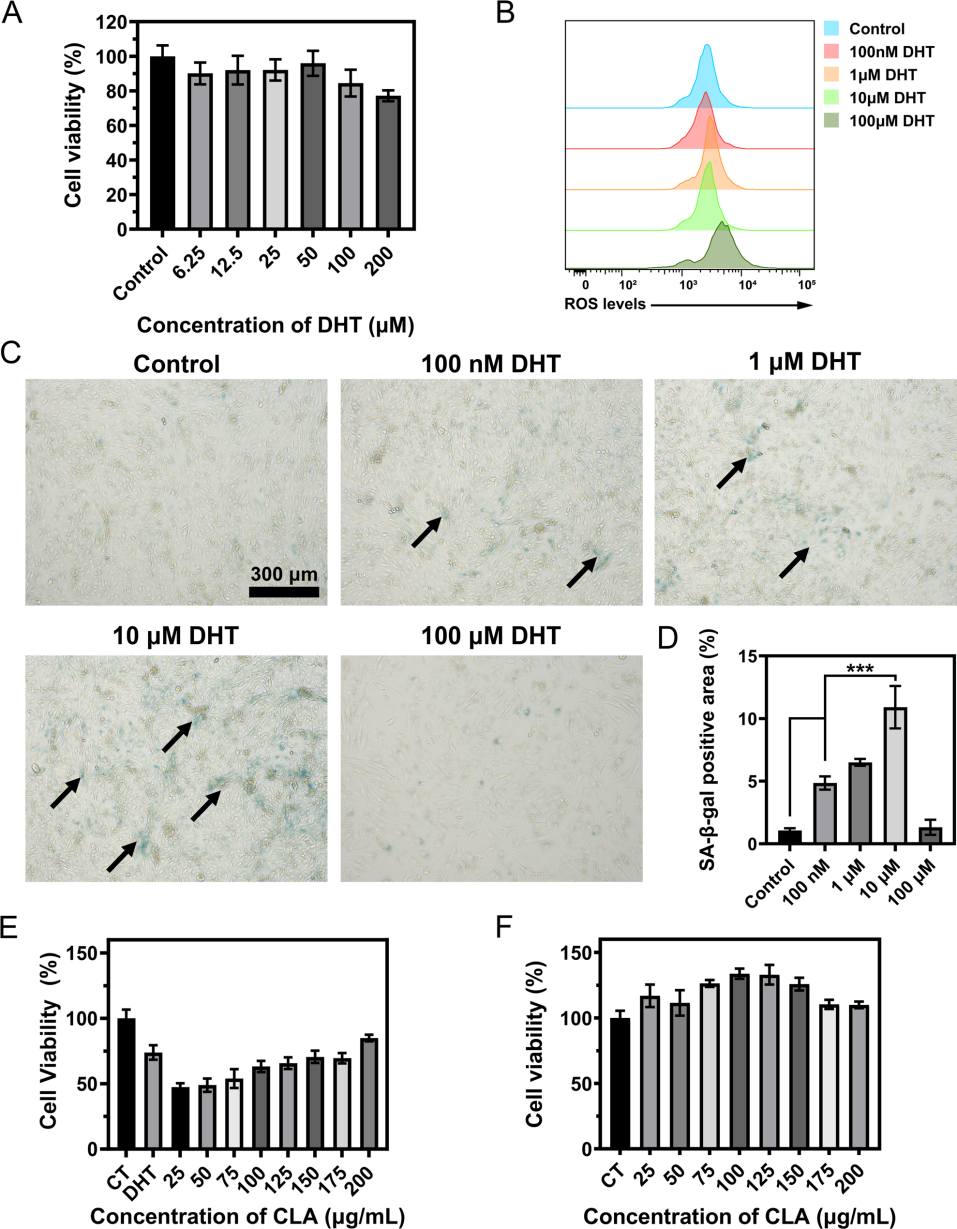
The effect on HDPCs cell viability (**A**), intracellular ROS level (**B**), SA-β-gal staining images (**C**), and quantitative analysis results of staining **(D**) under different concentrations of DHT. Black arrows: SA-β-gal positive points. The effects of on inhibiting cell damage induced by DHT (**E**) and promoting cell proliferation (**F**). ^***^P ˂ 0.001 vs. Control

Androgen/AR signaling was found to accelerate premature senescence in HDPCs, which is thought to reflect irreversible cell viability injury and cell growth arrest in the progression of AGA, thereby impairing their regenerative capacity and causing permanent damage to HFs [18]. Firstly, we detected how DHT affects the HDPCs’ viability, with results as shown in Fig. 2A. When DHT concentration was elevated to 200 µM, a marked reduction in cellular survival was observed. DHT also induced significant intracellular ROS (Fig. 2B). Followingly, we constructed a DHT-induced senescent cell model and employed β-galactosidase (marking SA-β-gal, the most characteristic marker of cellular senescence) staining to evaluate cellular senescence status [19]. As shown in Fig. 2C ~ 1D, a progressive increase in SA-β-gal-positive HDPCs was observed as DHT concentration was escalated from 100 nM to 10 µM. However, when the concentration was further elevated to 100 µM, marked morphological alterations and reduced cell counts indicated that cytotoxic effects predominated over senescence induction. This result highlighted that DHT triggered a reduction in HDPC viability and promoted an aging phenotype, and might even lead to cell death.

Meanwhile, we determined the modeling concentration for the subsequent cell experiments. A concentration of 200 µM was selected for induction of substantial cellular damage, where HDPCs viability was quantified as 77.22 ± 3.13%, and a concentration of 10 µM was established as optimal for inducing HDPCs aging, at which HDPCs had a positive SA-β-gal staining rate of 10.91 ± 1.69% without overt cytotoxicity.

Nutrient supplementation of PUFAs could promote hair regeneration *via* facilitating the telogen-anagen transition of HFs [20], while its role in inhibiting HFs aging is worthy of exploration. We evaluated the ability to inhibit the decline in cell viability induced by DHT of several PUFAs, including *ω*−3 (ALA, EPA, and DHA) and *ω*−6 (LA, CLA). The results of cell viability (Figure S1) showed that only CLA treatment saved the DHT-induced decline in cell viability (Fig. 2E ~ 1 F). The viability of HDPCs after PUFAs treatment was measured, and CLA exhibited the strongest pro-proliferative activity, with over 120% cell viability after treatment.

### Preparation and characterization of Arg-CLAVs

**Fig. 3:**
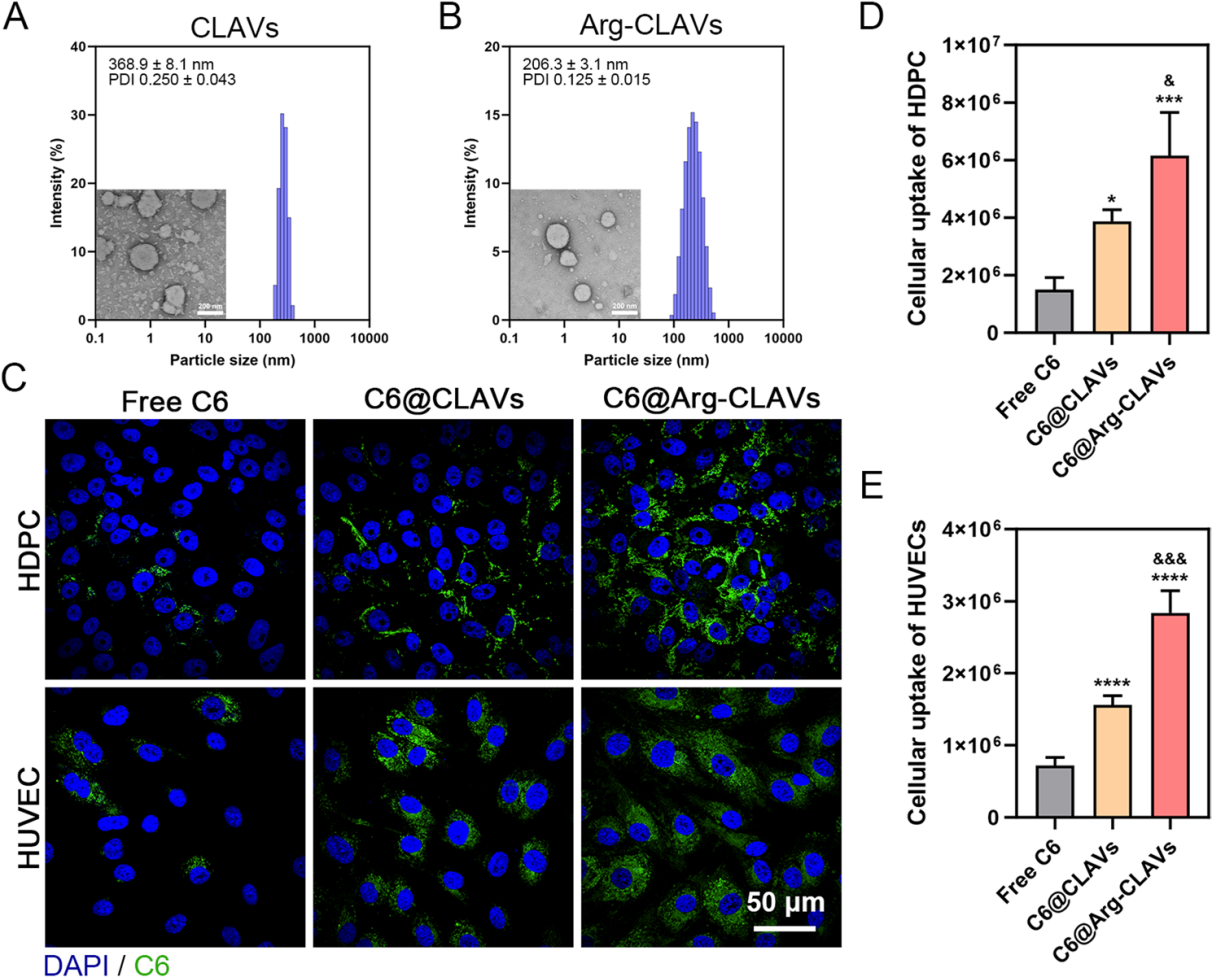
Characterizations of CLA-based nanovesicles. The particle size, size distribution, PDI, and TEM images of CLAVs (**A**) and Arg-CLAVs (**B**). The cellular uptake (**C**) and semi-quantitative results of C6@CLAVs and C6@Arg-CLAVs in HDPCs (**D**) and HUVECs (**E**). ^*^P ˂ 0.05, ^***^P ˂ 0.001, ^****^P ˂ 0.0001 vs. Control. ^&^*P* ˂ 0.05 vs. C6@CLAVs, ^&&&^*P* ˂ 0.001 vs. C6@CLAVs

CLA was found to self-assemble into stable vesicles due to its amphiphilicity, but only under alkaline conditions (pH 7.5 ~ 10.0), which is not suitable for application on the skin (pH 4.0 ~ 7.0). TPGS and EPC were introduced to co-assemble with CLA, enhancing stability across a broader pH range. The resulting co-assembled nanovesicles (CLAVs) exhibited good stability within an extended pH range of 6.0 to 8.0. CLAVs had a uniform particle size of 368.9 ± 8.1 nm and polydispersity index (PDI) of 0.250 ± 0.043 (Fig. 3A). Subsequently, CLAVs were functionalized with arginine (Arg-CLAVs), the particle size and PDI of which were 206.3 ± 3.1 nm and 0.125 ± 0.015, respectively (Fig. 3B). The morphologies of both nanovesicles were observed by TEM, exhibiting a relatively spherical shape (Fig. 3A ~ 2B). Then, FTIR was employed to further elucidate the mechanism of surface conjugation of Arg. As shown in Figure S2, compared with Arg, the stretching vibration peak of the amino group (3302 cm^− 1^) disappears, while a new peak appears at 3149 cm^− 1^, indicating a conversion of -NH_2_ group into -NH_3_^+^. The -COO^−^ stretching at 1630 cm^− 1^ emerged, accompanied by the peak intensity at 1741 cm^− 1^ markedly weakening. The result revealed that the surface conjugation of Arg-CLAVs was mediated through hydrogen-bonding interactions between the guanidinium/amino groups of Arg and the carboxyl groups of CLA. Besides, they demonstrated high stability over 90 days at both room temperature and 4 °C (Figure S3).

The positively charged guanidinium groups of Arg can interact with phosphate groups on cell membranes, triggering endocytosis through enhanced membrane affinity [21]. Therefore, a cellular uptake assay was arranged *by* detecting fluorescence of C6 encapsulated in Arg-CLAVs and CLAVs. Both HDPCs and HUVECs exhibited significantly higher fluorescence intensity in the Arg-CLAVs group compared to the CLAVs group (Fig. 3C ~ 2E). This enhanced cellular uptake efficiency substantiated that surface Arg-modified promoted rapid internalization of Arg-CLAVs, thereby accelerating payload delivery into target cells.

### Arg-CLAVs inhibited cell senescence

#### Arg-CLAVs alleviated DHT-induced cell damage and senescence in vitro

**Fig. 4:**
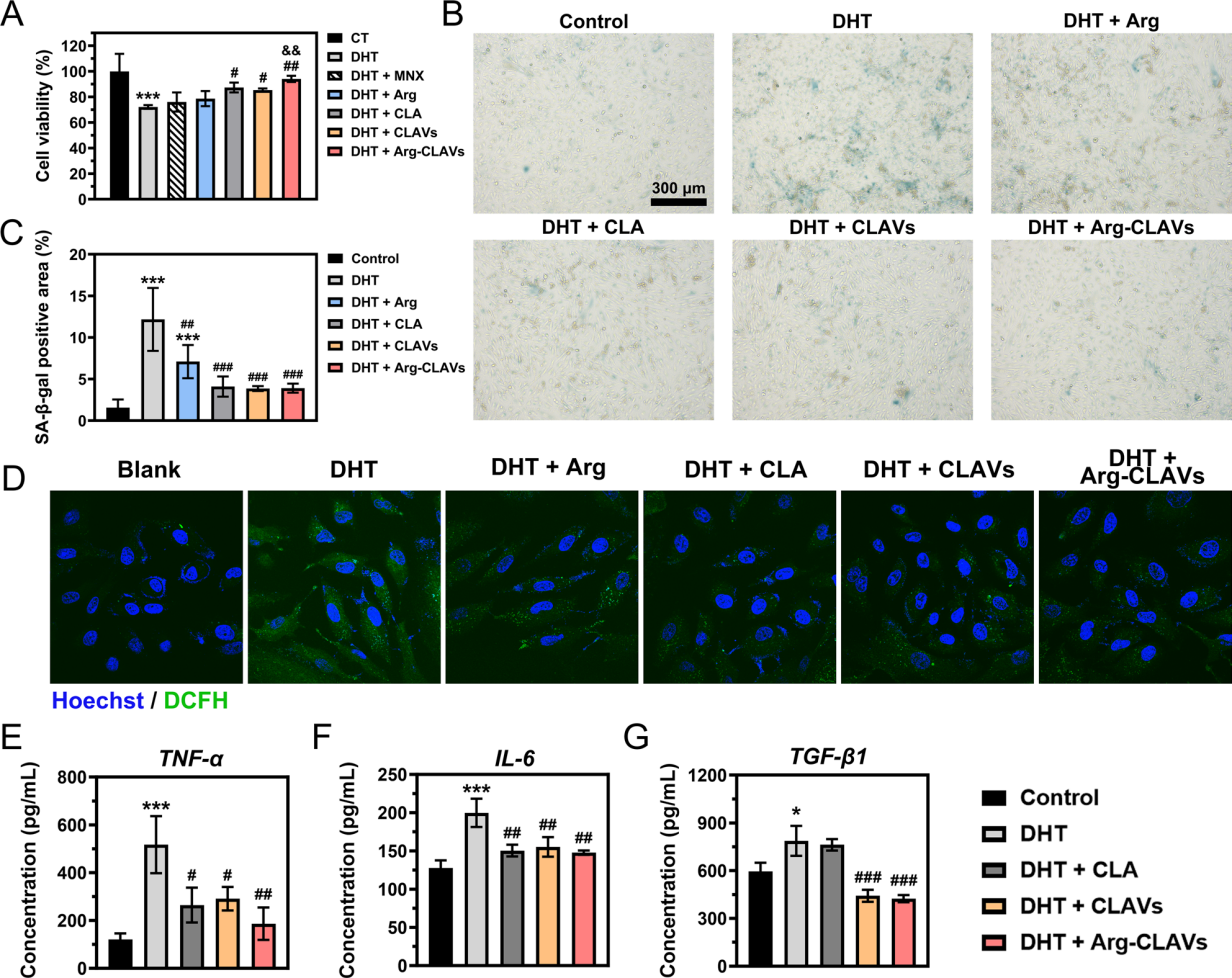
The anti-aging effect of Arg-CLAVs on HDPCs in vitro. (**A**) The cell viability of DHT-induced HDPCs after treatments. The SA-β-gal staining images (**B**) of DHT-induced HDPCs, and its staining positive rate (**C**). (**D**) Intracellular ROS of DHT-induced HDPCs captured by CLSM. The contents of SASP produced by HDPCs evaluated by ELISA kits: TNF-α (**E**), IL-6 (**F**), and TGF-β1 (**G**). ^*^P < 0.05, ^***^P < 0.001 vs. Control. ^#^*P* < 0.05, ^##^*P* < 0.01, ^###^*P* < 0.001 vs. DHT

The protective effects of different treatments on DHT-induced HDPCs injury are presented in Fig. 4A. Following DHT-induced injury, a significant reduction in HDPCs viability was observed, with values quantified as 72.13 ± 1.47%. Both MNX and Arg exhibited only slight protective effects on DHT-induced cellular damage. In contrast, significant improvements in cellular damage were observed in groups treated with CLA, CLAVs, and Arg-CLAVs, where HDPCs viability was restored to 87.38 ± 3.84%, 85.36 ± 1.31%, and 94.11 ± 2.43%, respectively. Notably, Arg-CLAVs demonstrated significantly superior efficacy compared to MNX in ameliorating HDPCs injury, thereby highlighting its therapeutic potential for reversing DHT-induced follicular cell damage.

Then, the degrees of cell aging after different treatments were determined with the positive rate of SA-β-gal staining (Fig. 4B ~ 3 C). Prolonged DHT exposure significantly induced senescence in HDPCs, leading to a marked elevation in SA-β-gal staining (12.18 ± 3.78%) compared with the negative control group (1.56 ± 0.99%). In the Arg group, the cell senescence induced by DHT was mildly relieved, and the staining positive rate decreased to 7.10 ± 2.00%, still significantly higher than that of the negative control group. After treatments with CLA, CLAVs and Arg-CLAVs, the number of senescent cells is significantly reduced, and the staining positive rate further dropped to 4.10 ± 1.22%, 3.86 ± 0.30%, and 3.91 ± 0.55% respectively, showing no significant difference from the negative control group.

In senescent cells, abnormal ROS accumulation induces mitochondrial damage and creates a vicious cycle of ROS overproduction, aggravating cellular aging [19]. Therefore, efficient removal of excessive ROS from senescent cells could potentially restore functional homeostasis and microenvironment stability, thereby alleviating age-related cellular deterioration.

DHT was used to stimulate senescence-associated oxidative stress in each treatment group. As presented in Fig. 4D, compared to the blank control group, HDPCs with only DHT-treatment exhibited abundant intracellular ROS production visualized through green fluorescence. Following Arg treatment, residual green fluorescence remained detectable within cells, indicating incomplete ROS clearance. In contrast, treatment with CLA, CLAVs, and Arg-CLAVs demonstrated marked ROS attenuation, particularly in the Arg-CLAVs group, where ROS signals were nearly undetectable.

We additionally employed ELISA kits to investigate three key SASP markers in skin and HFs, including TNF-α, IL-6, and TGF-β1 [22]. As shown in Fig. 4E ~ 3G, DHT treatment significantly increased the secretion levels of IL-6, TNF-α, and TGF-β1. CLA, CLAVs and Arg-CLAVs treatment all effectively downregulated the levels of both IL-6 and TNF-α, with Arg-CLAVs exhibiting the most inhibitory efficacy. CLAVs and Arg-CLAVs exerted the strongest inhibitory capacity against TGF-β1 production. These results indicated that Arg-CLAVs can effectively protect HDPCs against long-term damage caused by DHT, and reverse the aging state of HFs. These findings demonstrated that Arg-CLAVs effectively attenuated age-related HDPC damage, restrained DHT-induced SASP production and oxidative stress.

#### Arg-CLAVs remodeled the MAPK-ERK pathway in DHT-induced HFs aging

**Fig. 5:**
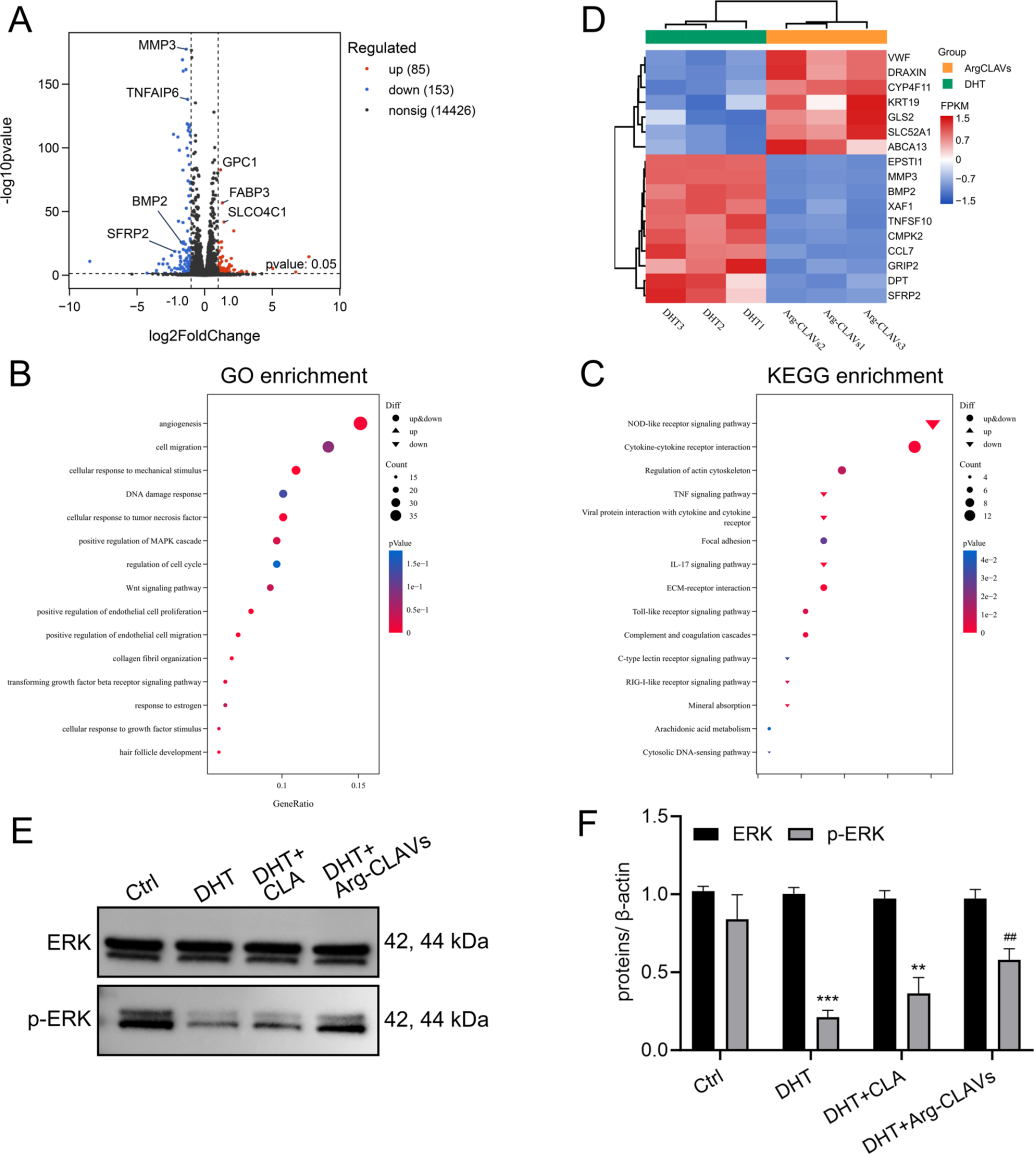
Arg-CLAVs remodeled the genes expression of DHT-treated HDPCs (*n* = 3). (**A**) The volcano plot of significantly upregulated genes (red) and downregulated genes (blue) between the two treatment groups. (**B**) The GO enrichment analysis. (**C**) The KEGG enrichment analysis. (**D**) The heat map of differentially expressed genes. (**E ~ F**) Western blots of HDPCs in different groups. ^**^P < 0.01, ^***^P < 0.001 vs. Control. ^##^*P* < 0.01 vs. DHT

To investigate the undergo molecular mechanism of Arg-CLAVs treatment on DHT-induced HFs, genome-wide expression profiles of HDPCs were examined by RNA-seq. Notably, the DHT-induced gene expression changes of HDPCs significantly differed from those of Arg-CLAVs treatment as indicated in the principal component analysis (PCA) (Figure S4), in which PC1 was 98.05% and PC2 was 0.78%. Along the PC1 axis, which represents the greatest source of variation, the DHT-treated (DHT) and Arg-CLAVs-treated (Arg-CLAVs) samples were completely separated. All three Arg-CLAVs-treated replicates clustered tightly in the negative region of PC1, while all three replicates treated with DHT clustered in the positive region. This clear separation demonstrates that the Arg-CLAVs treatment is the dominant driver of gene expression differences in this dataset. As shown in the volcano plot, a total of 85 differentially expressed genes (DEGs) were up-regulated while 153 were down-regulated (Fig. 5A), with notable alterations in genes related to angiogenesis, cell migration, cellular response to TNF, regulation of cell cycle and Wnt signaling pathways (Fig. 5B). The NOD-like receptor signaling pathway was markedly down-regulated in Arg-CLAVs-treated HDPCs (Fig. 5C). A gene clustering heat map was created by selecting representative genes involved in the HFs cycle and aging (Fig. 5D). Some genes inversely correlated with hair regeneration, such as bone morphogenetic protein 2 (BMP2) and matrix metalloproteinase 3 (MMP3), were downregulated by Arg-CLAVs treatment, meanwhile other genes that indirectly affect hair generation also came down, such as epithelial stromal interaction 1 (EPSTI1, related to activating TGF-β pathway and IL-6/STAT3 pathway). The transcriptomic analysis implied that Arg-CLAVs could alleviate cell aging in HDPCs exposed to DHT. Of note, gene set enrichment analysis (GSEA) further revealed significant DHT vs. Arg-CLAVs upregulation of the MAPK pathway (Figure S5A). Gene Ontology (GO) analysis indicated that Arg-CLAVs treatment could inhibit cell aging *via* MAPK-ERK signaling (Figure S5B). Using western blots of DHT-induced HDPCs, strongly upregulated p-ERK was detected in Arg-CLAVs-treated HDPCs (Fig. 5E ~ 5 F).

We also used RT-qPCR to detect the expression levels of key genes associated with HF aging in HDPCs, including *SRD5A2*, *CDKN2A*, and *p53* [23]. CLA and Arg-CLAVs treatments significantly downregulated the elevated mRNA expression of *SRD5A2*,* CDKN2A*, and *p53* led by DHT, indicating that Arg-CLAVs might inhibit the cell aging progression (Figure S6A ~ S6C).

Accordingly, the effects of Arg-CLAVs on positive genes in hair generation were also detected. Wnt/β-catenin signaling pathway plays a pivotal role as a critical positive regulator regulating HFs anagen, while its significant suppression is characteristically observed in AGA [23]. *FGF-7* could activate quiescent HFSC [24], and *IGF-1* is a key nutrient-sensing factor in skin and HFs, impairing cellular nutrient uptake capacity [22]. Treatments with CLA and Arg-CLAVs remarkably upregulated the mRNA expression of *β-Catenin* affected by DHT, wherein Arg-CLAVs achieved nearly complete normalization compared to the negative control group (Figure S6D). As shown in Figure S6E ~ S6F, CLA and Arg-CLAVs significantly upregulated the mRNA levels of *FGF-7* and *IGF-1* in HDPCs treated with DHT, even exceeding levels observed in the negative control group. Taken together, these implied that Arg-CLAVs could prevent the aging of HFs by inhibiting SASP production and regulating hair generation-related gene expression.

### Arg-CLAVs remodeled the HF niche in vitro

The homeostatic balance of the HF niche is a fundamental determinant in both sustaining normal growth cycles and preventing the HF senescence. Early-stage follicular miniaturization in AGA has been recognized as associated with the breakdown of the HF niche, characterized by an atrophic capillary network impairing the nutritional support for HFs, and microenvironmental oxidative burden causing cell dysfunction [23, 25]. In addition, the proliferation of DPCs is also critically important, representing a key process for anagen. Therefore, we investigated the abilities of Arg-CLAVs to induce vascularization, remove oxidative pressure, and modulate the proliferation and migration of HDPCs.

**Fig. 6:**
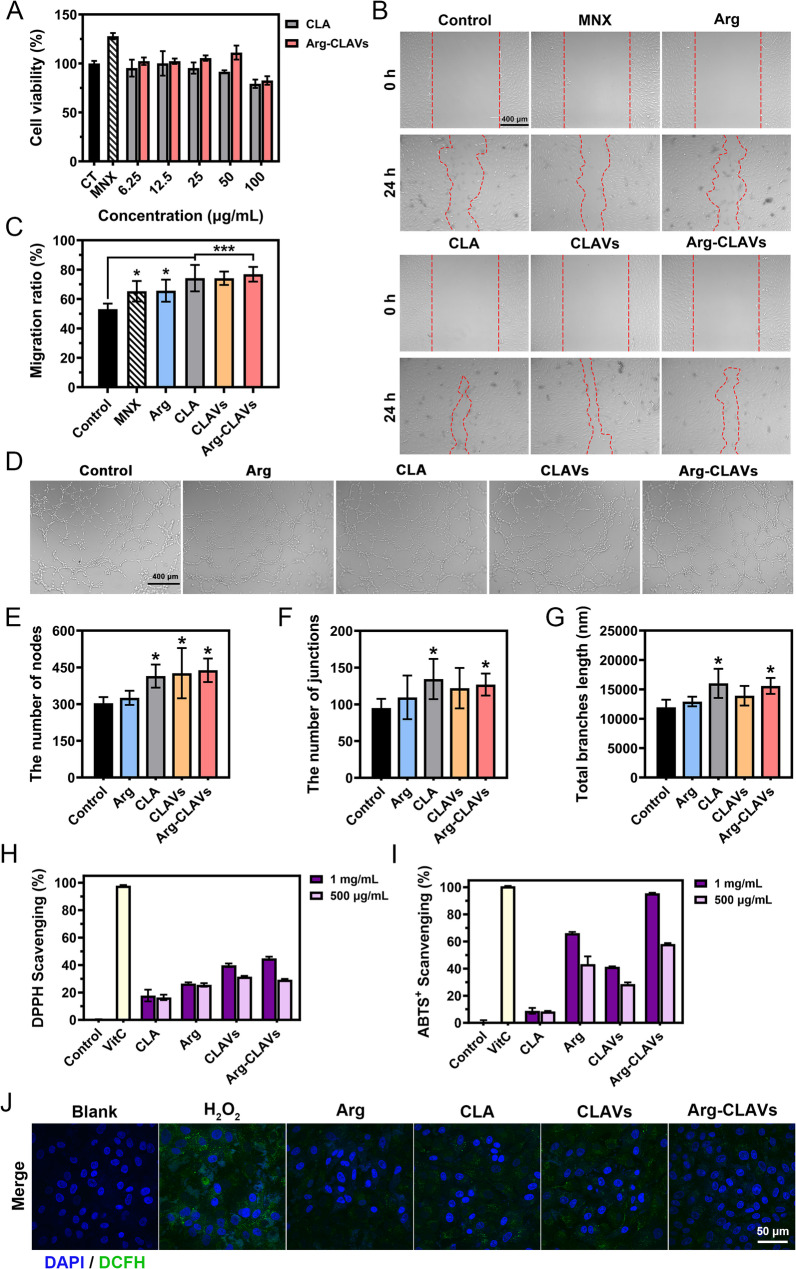
Arg-CLAVs promote proliferation, migration, and in vitro tube formation of HUVECs. (**A**) The effect on HUVECs cell viability under different concentrations of CLA and Arg-CLAVs. The effect of different groups on HUVECs migration: representative photos** (B**) and quantitative analysis results (**C**). The effect of different groups on HUVECs tube formation in vitro: representative photos (**D**) and quantitative analysis (**E ~ G**). The scavenging effect of different treatments on DPPH (**H**) and ABTS^+^ (**I**) free radicals. (**J**) The scavenging effect of different treatments on intracellular ROS of HDPCs induced by H_2_O_2_ was captured by CLSM. ^*^P ˂ 0.05, ^***^P ˂ 0.001 vs. Control

As illustrated in Fig. 6A, within the concentration range of 6.25 ~ 50 µg/mL, Arg-CLAVs demonstrated concentration-dependent pro-proliferative activity on HUVECs, having cell viability as high as 111.14 ± 7.12% at 50 µg/mL, which might contribute to the bioactivity of Arg because there was no difference in HUVECs viability between the CLA treatment group and the control group. Arg has been proven to stimulate vascular regeneration as a nitric oxide precursor, by converting to nitric oxide *via* nitric oxide synthase activity, subsequently activating endothelial-dependent signaling pathways and driving neovascularization [26]. As for the effect on enabling HUVECs migration (Fig. 6B ~ 5 C), the CLA, CLAVs, and Arg-CLAVs treatments narrowed scratch gaps significantly, with the migration rates of 74.22 ± 9.00%, 74.17 ± 4.53%, and 76.86 ± 5.01%, respectively. In contrast, the Arg group and the MNX group showed relatively lower migration rates than CLA and CLAVs (65.70 ± 7.50% and 65.32 ± 7.02%), though statistically higher than the control group (53.11 ± 3.80%). The in vitro tube-formation assay further demonstrated the pro-angiogenic potential of Arg-CLAVs. Compared to the control group, the CLA group, the CLAVs group and the Arg-CLAVs group significantly enhanced the formation of capillary-like networks (Fig. 6D). Quantitative analysis revealed that the total branch length as well as the number of nodes and junctions of CLA and Arg-CLAVs treated groups demonstrated superior performance than the control group (Fig. 6E ~ 5G).

The activities of removing the oxidative burden of Arg-CLAVs were evaluated by radical scavenging assay and intracellular ROS scavenging assay. Arg-CLAVs at a concentration of 1 mg/mL demonstrated a DPPH scavenging rate of 44.96 ± 1.18% (Fig. 6H). As for ABTS⁺ radical elimination, CLA showed limited efficacy at 1 mg/mL (8.84 ± 2.14%), while Arg achieved 66.09 ± 0.92% scavenging rates at the same concentration. Interestingly, the antioxidant performance of CLAVs was enhanced compared to CLA. This might be attributed to the fact that TPGS and EPC provide additional antioxidant action. Moreover, Arg-CLAVs achieve an exceptional ABTS⁺ elimination rate of 95.53 ± 0.42% at 1 mg/mL (Fig. 6I), implying cooperative activity of Arg with CLA. As illustrated in Fig. 6J, H_2_O_2_ was utilized to stimulate the exogenous oxidation, which triggered intense green fluorescence in the cells versus the blank control, indicating substantial ROS generation. Remarkably, the Arg-CLAVs group exhibited the most effective ROS clearance, with minimal fluorescence detected post-treatment, which could contribute to co-antioxidant activity from Arg and CLA combined with improved cellular uptake.

**Fig. 7:**
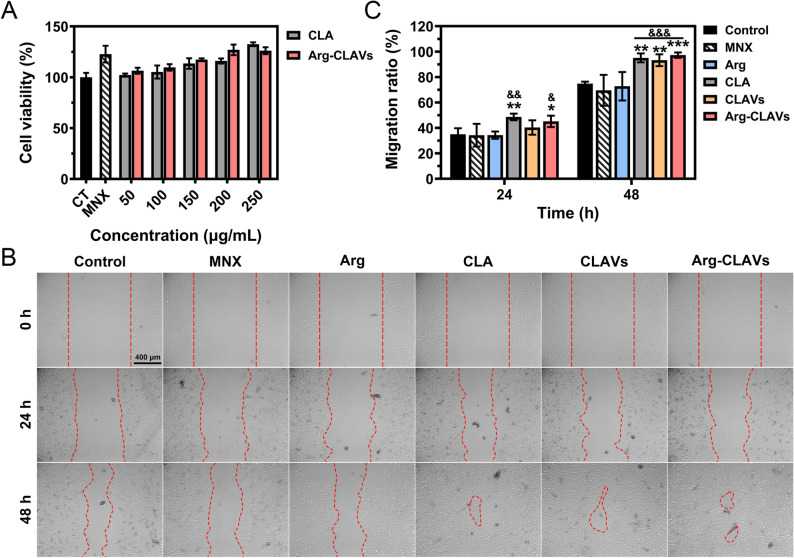
Arg-CLAVs promoted the proliferation and migration of HDPCs. (**A**) The effect on HDPCs cell viability under different concentrations of CLA and Arg-CLAVs. The effect of different groups on HDPCs migration: representative photos (**B**) and quantitative analysis results **(C**). ^*^P ˂ 0.05, ^**^P ˂ 0.01, ^***^P ˂ 0.001 vs. Control. ^&^*P* ˂ 0.05, ^&&^*P* ˂ 0.01, ^&&&^*P* ˂ 0.001 vs. MNX

Then, we investigated the effects on HDPCs of Arg-CLAVs on cell viability and migration ability. Both CLA and Arg-CLAVs could promote HDPCs proliferation in a concentration-dependent manner from 50 to 200 µg/mL (Fig. 7A). Especially, the viability of HDPCs in Arg-CLAVs was as high as 126.99 ± 5.21% at 200 µg/mL, exceeding that of MNX (122.78 ± 8.21%). Subsequently, the migration ability of HDPCs was analyzed *via* scratch assays. As shown in Fig. 7B ~ 6 C, scratch gaps in the CLA and Arg-CLAVs groups were significantly narrower than in the control and MNX groups, with migration rates of 48.65 ± 2.65%, 45.14 ± 4.49%, 35.05 ± 4.74%, and 34.29 ± 8.94%, respectively at 24 h. By 48 h, near-complete scratch closure was observed in the CLA, CLAVs, and Arg-CLAVs groups. The migration rates of the three groups were 95.07 ± 3.52%, 93.29 ± 4.67% and 97.28 ± 2.11%, respectively, all superior to that of the control group (74.78 ± 1.63%) and the MNX group (69.60 ± 12.16%).

### Skin penetration, retention, and follicular targeting behavior of Arg-CLAVs

MNX, a topical vasodilator, acts through its active metabolite (minoxidil sulfate), which enhances perifollicular blood flow by dilating microvessels. It also could prolong the HF anagen by upregulating the Wnt/β-catenin pathway and some beneficial factors (e.g. VEGF, IGF-1, and FGF-2). However, these are insufficient to address the issue of HF aging. Here, MNX was added into Arg-CLAVs to prepare MNX-loaded nanovesicles (MNX@Arg-CLAVs), therefore broadening its application in anti-HF aging. MNX@Arg-CLAVs had uniform particle (size of 206.0 ± 2.9 nm, and PDI of 0.136 ± 0.008, Figure S7), efficient entrapment of MNX (EE of 97.14 ± 1.89%, and DL of 4.86 ± 0.13%, Table S2), and high stability (Figure S3). Notably, the organic solvent content in MNX@Arg-CLAVs prescription was substantially lower than that in tinctures, and it didn’t include propylene glycol (Table S3).

**Fig. 8:**
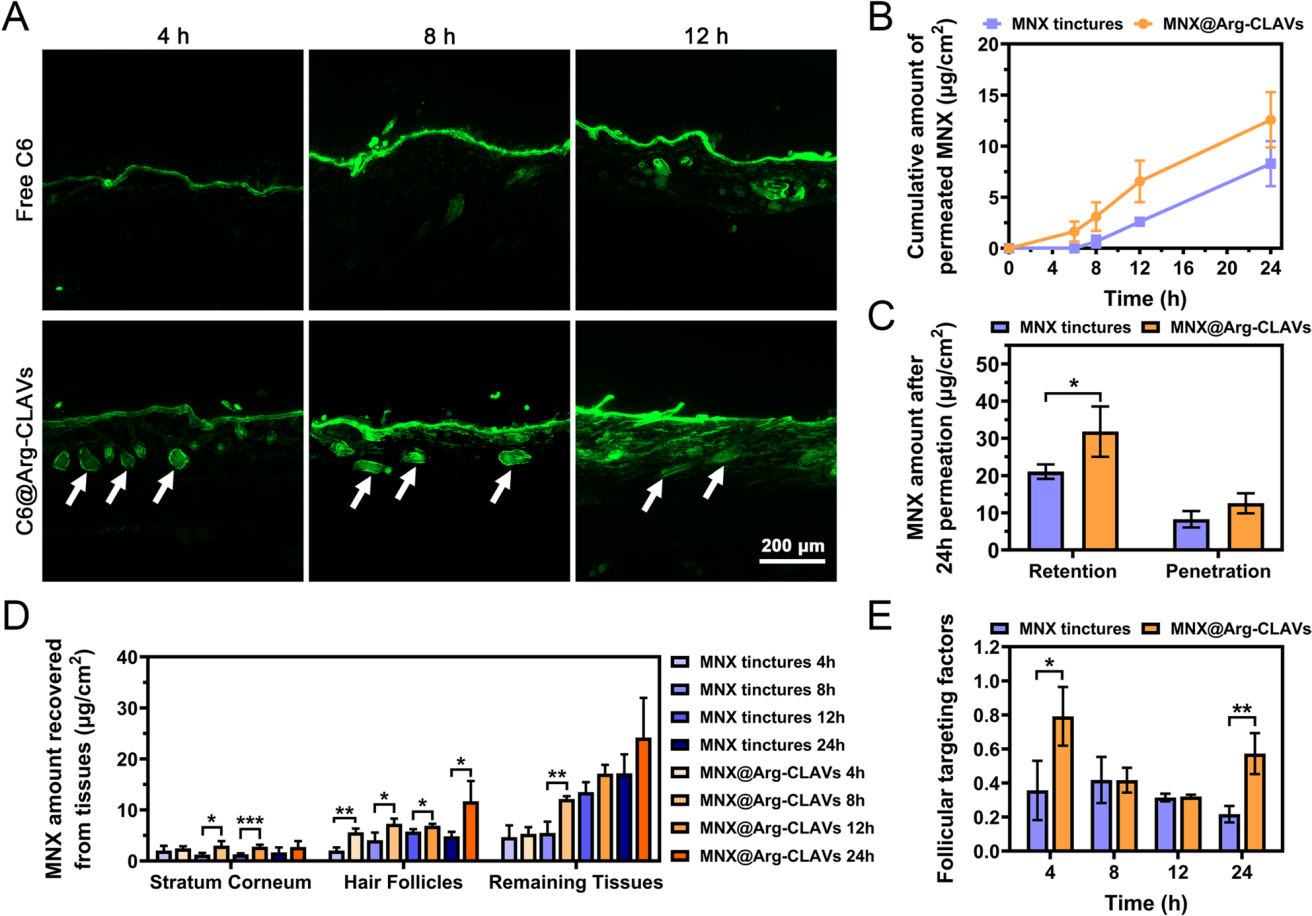
Transdermal behaviors and hair follicles targeting effect of Arg-CLAVs. (**A**) Skin penetration of free C6 and C6@Arg-CLAVs in rat skin at different time points (white arrows: hair follicles). Bar = 200 μm (**B**) The cumulative amount of MNX penetrating porcine skin in MNX tinctures and MNX@Arg-CLAVs. (**C**) The MNX amount of skin retention and penetration in MNX tinctures and MNX@Arg-CLAVs after 24 h. The MNX amount in different skin parts (**D**) and the follicular targeting factors (**E**) of MNX tinctures and MNX@Arg-CLAVs. ^*^P ˂ 0.05, ^**^P ˂ 0.01, ^***^P ˂ 0.001

First of all, we evaluated the skin permeation behavior of Arg-CLAVs and MNX@ Arg-CLAVs on ex vivo abdominal skin of rats. Arg-CLAVs loading C6 (C6@Arg-CLAVs) was constructed for visualizing the HF-targeting behavior of Arg-CLAVs in vitro. As shown in Fig. 8A, at 4 h post-treatment, the free C6 group exhibited weak fluorescence localized in the stratum corneum, while the C6@Arg-CLAVs group had more accumulation in both HFs and the stratum corneum, showing its potential for HF targeting. This might be attributed to the “ratchet effect” of nanocarriers. That is, the nanocarriers are physically entrapped by keratin of the hair shaft during hair movement and subsequently transported deep into the HFs [27]. After 8 h of treatment, progressive fluorescence enhancement was observed in both groups. However, most of the fluorescence of the free C6 group remained in the stratum corneum with minimal accumulation at HFs. In contrast, the green fluorescence of the C6@Arg-CLAVs group was strengthened and spread with the help of nanovesicles. By 12 h, the free C6 gathered moderately with HFs but maintained less weak in the dermal layer. Conversely, the C6@Arg-CLAVs had penetrated deep into the dermis with lasting fluorescence within HFs. These findings revealed that the nanovesicle was a promising carrier for topical drug delivery systems due to its follicular targeting and stratum corneum permeation enhancement.

Furthermore, the skin penetration, retention, and follicular targeting behaviors were monitored on ex vivo abdominal skin of the Bama pig, and quantitatively analyzed using HPLC by detecting the MNX content. The diluent commercial MNX tincture with the same MNX concentration as MNX@Arg-CLAVs served as the control group. The result showed that, in the MNX@Arg-CLAVs group, MNX could be detected as early as 6 h, whereas that of the MNX tincture group was not detected until 8 h. After 12 h, the cumulative amount of permeated MNX in the MNX@Arg-CLAVs reached 12.58 ± 2.72 µg/cm², overpassing 8.29 ± 2.21 µg/cm² of that in the MNX tincture group (Fig. 8B), demonstrating the better skin penetration efficacy of the nanovesicles. Moreover, the nanovesicles were able to form drug reservoirs within the skin, possessing sustained release and long-term therapeutic ability. Compared to the commercial MNX tincture, MNX@Arg-CLAVs group retained in the skin was 31.79 ± 6.77 µg/cm^2^ after 12 h of application, exceeding 21.07 ± 1.90 µg/cm^2^ detected in the MNX tincture group (Fig. 8C), implying the MNX@Arg-CLAVs not only could permeate skin faster but also displayed significant retention ability.

Meanwhile, to know how MNX was encapsulated in two formulations distributed within the skin, tape stripping-cyanoacrylate biopsy analysis was deployed to quantify the MNX content in the different cutaneous structures [28]. As illustrated in Fig. 8D ~ 7E, the higher content of MNX was accumulated in the HFs (5.64 ± 0.71 µg/cm²) compared to the stratum corneum (2.48 ± 0.41 µg/cm²) and residual skin tissues (5.33 ± 1.33 µg/cm²) at 4 h post-application. The follicular targeting factor, defined as the ratio of follicular MNX content to the total cutaneous MNX deposition [29], reflects the ability of carriers delivering drug to HFs more accurately, which of the MNX@Arg-CLAVs group (0.79 ± 0.17) significantly surpassed that of the MNX tincture group (0.36 ± 0.17) at the fourth hour. All these outcomes indicated that MNX@Arg-CLAVs localized to HFs as soon as application, exhibiting obvious follicular targeting action. Followingly, the MNX levels in surrounding skin compartments increased and the follicular targeting factor declined gradually, because nanovesicles spread out from HFs to the surrounding tissues. However, the amount of the nanovesicle accumulating within the HFs still remained much higher than that of the tincture (Fig. 8D). After 24 h, the follicular targeting factor of MNX@Arg-CLAVs (0.57 ± 0.12) significantly elevated in comparing with that of tincture (0.22 ± 0.05) (Fig. 8E). It might stem from the better retention capacity of MNX@Arg-CLAVs at HFs, while lots of MNX in tincture was rapidly cleared from both skin and HFs.

Collectively, MNX encapsulated in Arg-CLAVs exhibited rapid cutaneous delivery, spatial-temporal controlled release and prolonged follicular accumulation.

### Assessment of Arg-CLAs promoting hair growth on AGA model

#### In vivo efficacy on male AGA mice

**Fig. 9:**
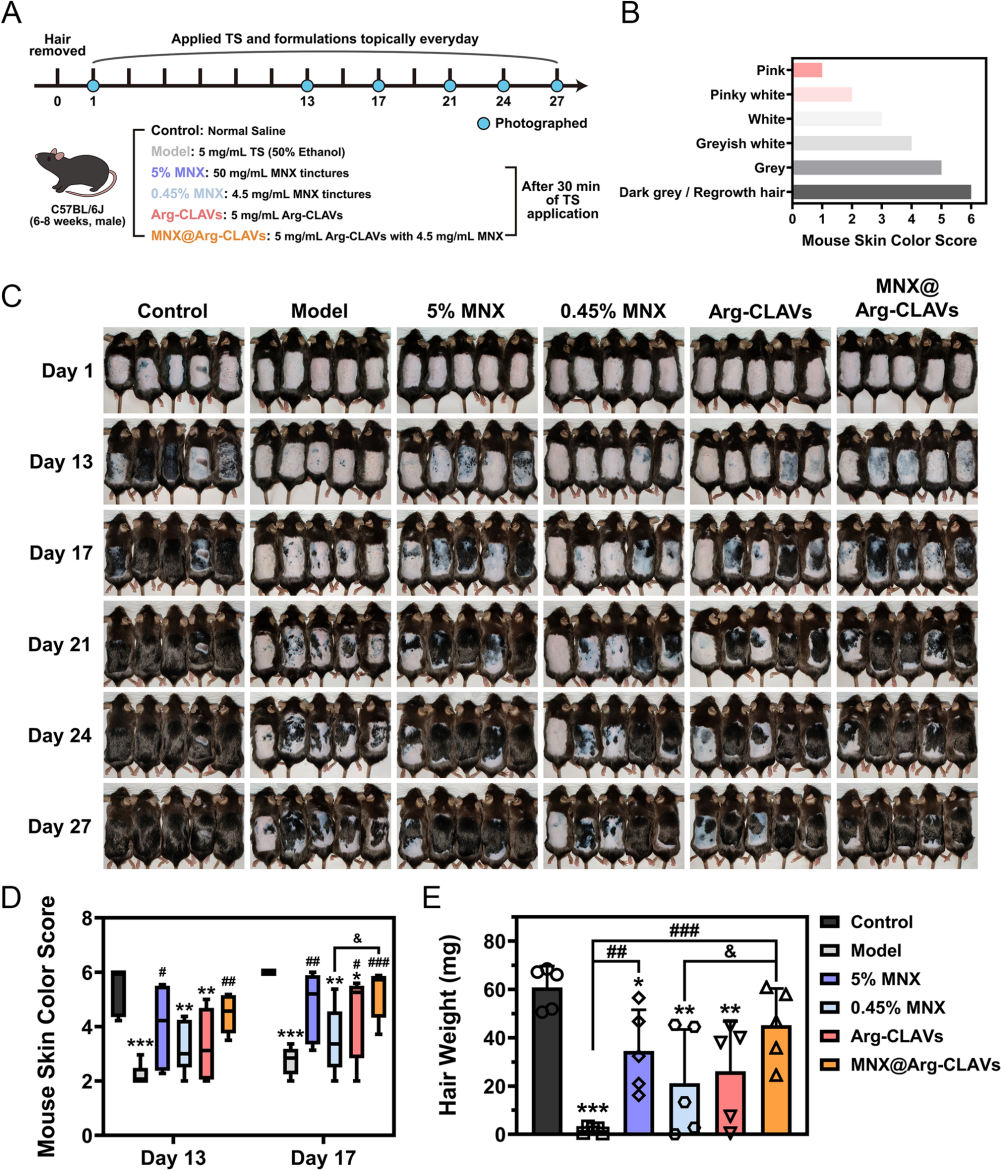
In vivo efficacy evaluation of nanovesicles on AGA male model. (**A**) Treatment schedule. (**B)** The rating scale of mouse skin color. (**C**) Photographs of hair growth. The mouse skin color score (**D**), and the weight of regenerated hair (**E**). ^*^P ˂ 0.05, ^**^P ˂ 0.01, ^***^P ˂ 0.001 vs. Control. ^#^*P* ˂ 0.05, ^##^*P* ˂ 0.01, ^###^*P* ˂ 0.001 vs. Model. ^&^*P*˂ 0.05 vs. 0.45% MNX

To investigate the efficacy of Arg-CLAVs on inducing hair growth in vivo, C57BL/6J mice were treated with TS topically to establish an AGA model. The experiment protocol was outlined in Fig. 9A. The skin color scoring standard was shown in Fig. 9B. As shown in Fig. 9C, the control group exhibited significant hair regrowth by day 13, with complete dorsal hair coverage observed by day 17. In contrast, model group mice manifested delayed skin pigmentation changes, displaying only partial hair regrowth at day 27, thereby validating successful AGA induction. Compared to the model group, treatments with 5% MNX, Arg-CLAVs, and MNX@Arg-CLAVs induced early skin pigmentation by day 13, indicating the HFs were transiting from telogen to anagen. All treated groups achieved superior skin color scores on day 17 compared to the model group (Fig. 9D), with MNX@Arg-CLAVs demonstrating the toppest score, and significantly higher than both 5% MNX tincture. Notably, the variability of the skin color score of mice in 5% MNX group was relatively high, which indicated that the therapeutic efficacy of 5% MNX tincture varies greatly among individuals [30]. Interestingly, individual variability was lower in the MNX@Arg-CLAVs group. By day 27, complete hair regrowth was observed in both 5% MNX and MNX@Arg-CLAVs groups, while Arg-CLAVs and 0.45% MNX groups showed partial hair coverage. Moreover, MNX@Arg-CLAVs group demonstrated the greatest hair mass weight (45.17 ± 15.22 mg), significantly exceeding the model group (1.80 ± 1.28 mg), 0.45% MNX group (21.17 ± 22.21 mg), and even commercial 5% MNX tincture (34.55 ± 17.00 mg) (Fig. 9E). This suggests that MNX@Arg-CLAVs, despite containing only 0.45% MNX, achieved superior therapeutic efficacy rather to commercial tincture, and contained significantly reduced organic solvent content, thereby minimizing skin irritation [15] associated with high-concentration propylene glycol.

**Fig. 10:**
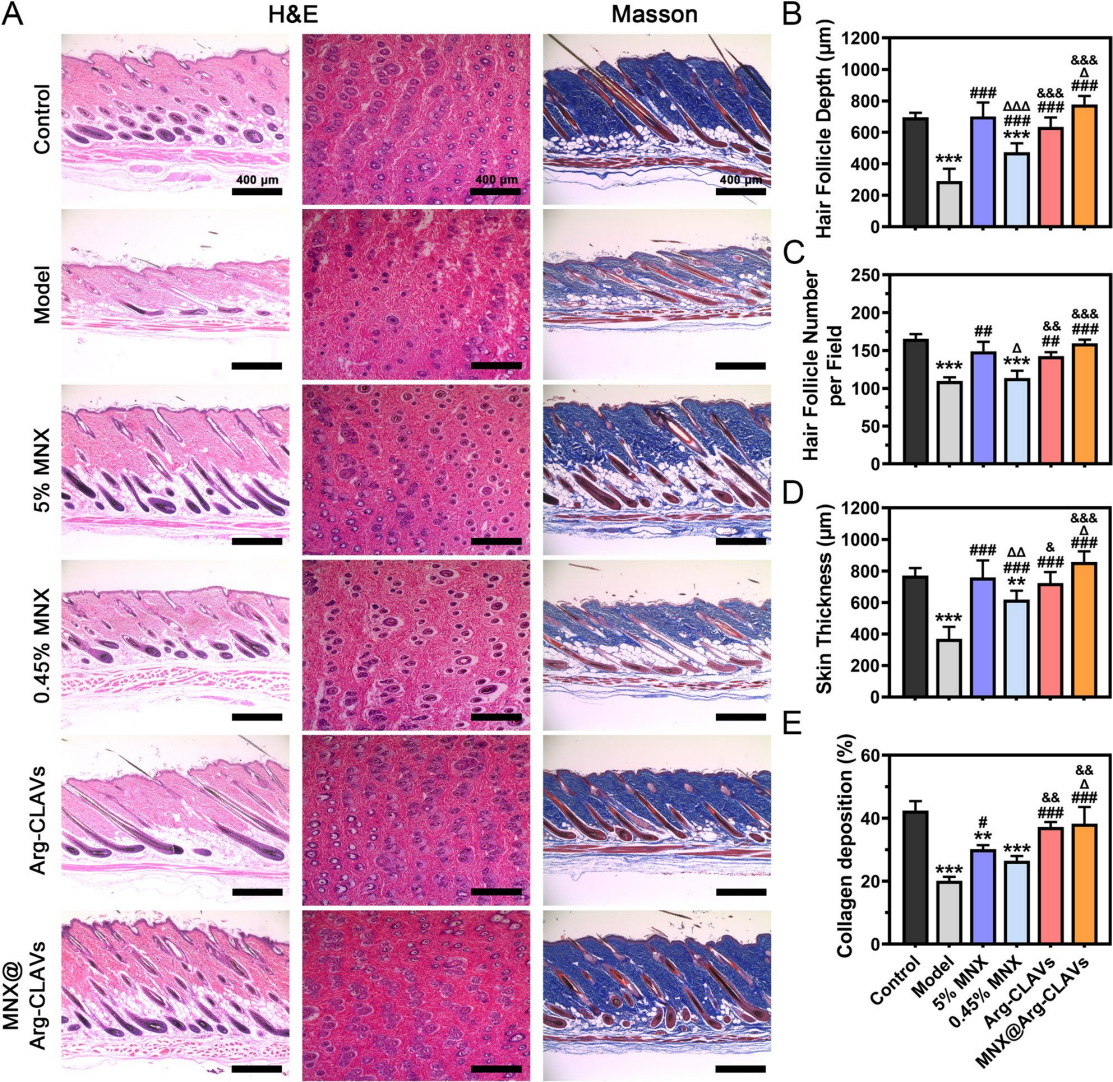
The H&E staining and Masson staining of vertical skin, horizontal skin (**A**), the hair follicle depth (**B**), the number of hair follicles per field (**C**), skin thickness (**D**), and collagen deposition **(E**) in male AGA model treated by different formulations. ^**^P ˂ 0.01, ^***^P ˂ 0.001 vs. Control. ^#^*P* ˂ 0.05, ^##^*P* ˂ 0.01, ^###^*P* ˂ 0.001 vs. Model. ^Δ^*P* ˂ 0.05, ^ΔΔ^*P* ˂ 0.01, ^ΔΔΔ^*P* ˂ 0.001 vs. 5% MNX. ^&^*P* ˂ 0.05, ^&&^*P* ˂ 0.01, ^&&&^*P* ˂ 0.001 vs. 0.45% MNX. Bar = 400 μm

Histopathological evaluation *via* H&E staining (Fig. 10A) revealed that the control group exhibited abundant follicles with bulbous DP, while the model group showed representative AGA characteristics characterized by follicular miniaturization and DP atrophy. The MNX@Arg-CLAVs group exhibited increased follicular density and restored DP morphology. Quantitative analysis of follicular depth (Fig. 10B) demonstrated that of the HFs in both 5% MNX and MNX@Arg-CLAVs groups grew downward into the dermis and deep subcutis. Transverse skin sections were analyzed to quantify the number of regenerative HFs (Fig. 10C). The model group exhibited significantly reduced HFs density (109.7 ± 4.93 follicles/mm²) compared to controls, accompanied by remarkable follicle miniaturization. MNX@Arg-CLAVs group achieved the highest follicle count (159.7 ± 4.73 follicles/mm²), followed by 5% MNX (149.0 ± 12.53 follicles/mm²) and Arg-CLAVs (142.7 ± 5.13 follicles/mm²), while the 0.45% MNX group remained the minimum follicular count (113.7 ± 9.61 follicles/mm²).

Cellular senescence in AGA induces massive release of SASP components, which further degrade collagen fibers through matrix metalloproteinase activity, thereby causing the thinning of skin and compromising the support and growth of the HFs. Therefore, collagen deposition was evaluated through Masson’s trichrome staining to assess skin matrix remodeling. As shown in Fig. 10B ~ 9E, the model group presented the thinnest skin along with the least collagen staining, indicating TS induction led to a reduction of both skin thickness and collagen deposition. Although 5% MNX and 0.45% MNX treatments promoted hair growth and restored skin thickness, they were ineffective in improving collagen degradation. In contrast, MNX@Arg-CLAVs treatment successfully restored both skin thickness and collagen deposition to near-normal levels.

**Fig. 11:**
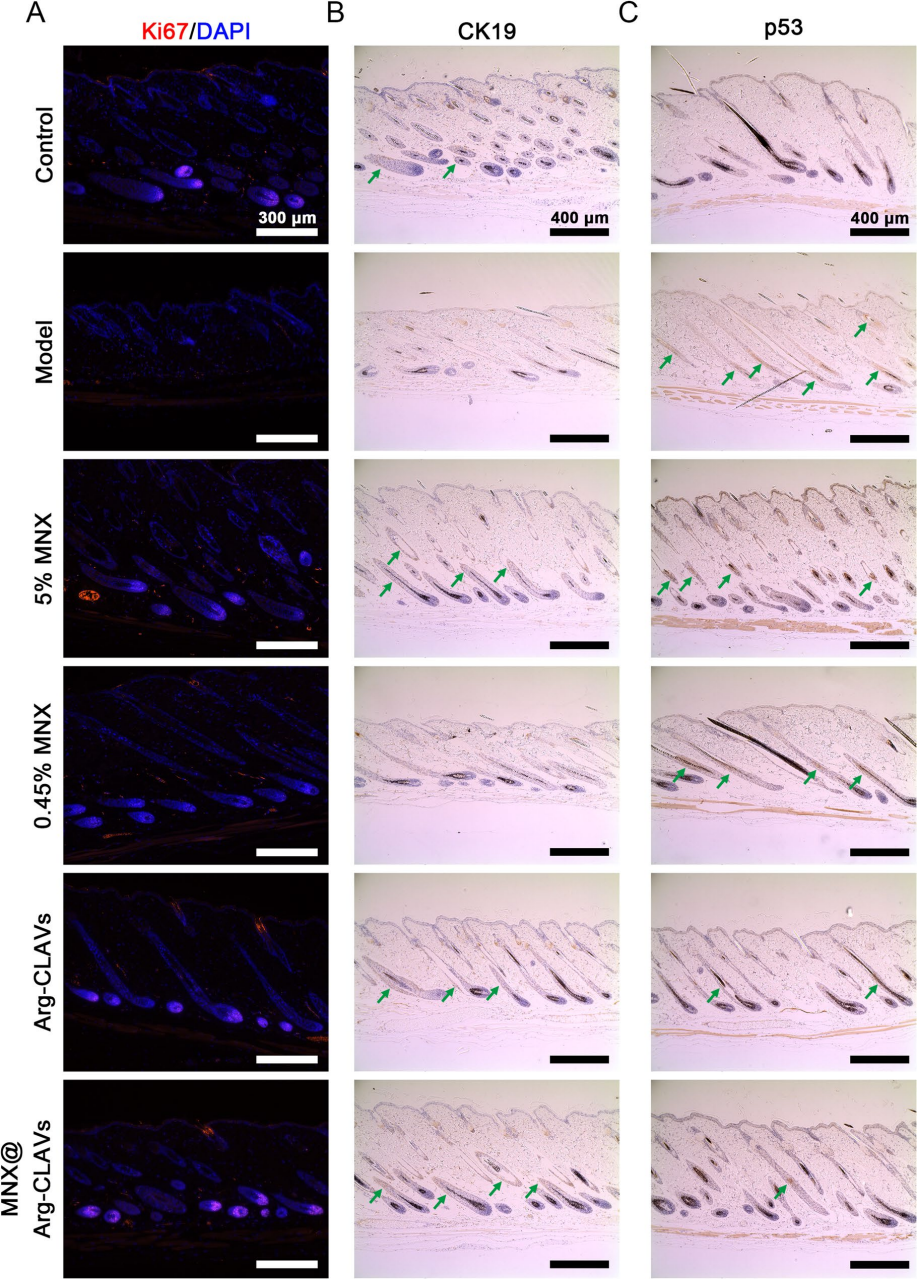
The immunofluorescent staining of Ki67 (**A**) and the immunohistochemical staining of CK19 (**B**) and *p53* (**C**) in male AGA model skins treated by different formulations (Green arrows: CK19, *p53* positive) (bar in A = 300 μm, bar in B and C = 400 μm)

As MNX@Arg-CLAVs can induce anagen, the anagen-related markers, including Ki67 (cellular proliferation) and CK19 (HFSC activation), showed the highest expression in MNX@Arg-CLAVs treated mice (Fig. 11A ~ 10B), further suggesting active HF growth after treatment. We also measured the expression of *p53* in the dorsal skin of treated mice (Fig. 11C). Compared with the control group, *p53* in the model group was significantly expressed, also indicating that AGA mice had the characteristic of HF senescence. Notably, strong *p53* positivity was still observed in both 0.45% MNX and 5% MNX groups, which meant they failed to improve cell senescence. On the other hand, Arg-CLAVs and MNX@Arg-CLAVs groups significantly lowered *p53* levels, implicating the potential of Arg-CLAVs as a nanoplatform synergizing with other drugs to alleviate HF senescence and promote hair growth.

#### In vivo efficacy on female AGA mice

Despite the similarity in the pathogenic mechanism of AGA between genders, the progression of this disease differs, which in turn leads to variations in the therapeutic effects of medications [1]. In order to investigate whether MNX@Arg-CLAVs have consistent efficacy across gender-specific AGA models, female AGA mice were subjected to identical modeling protocols, followed by evaluation of treatment outcomes (Figure S8).

**Fig. 12:**
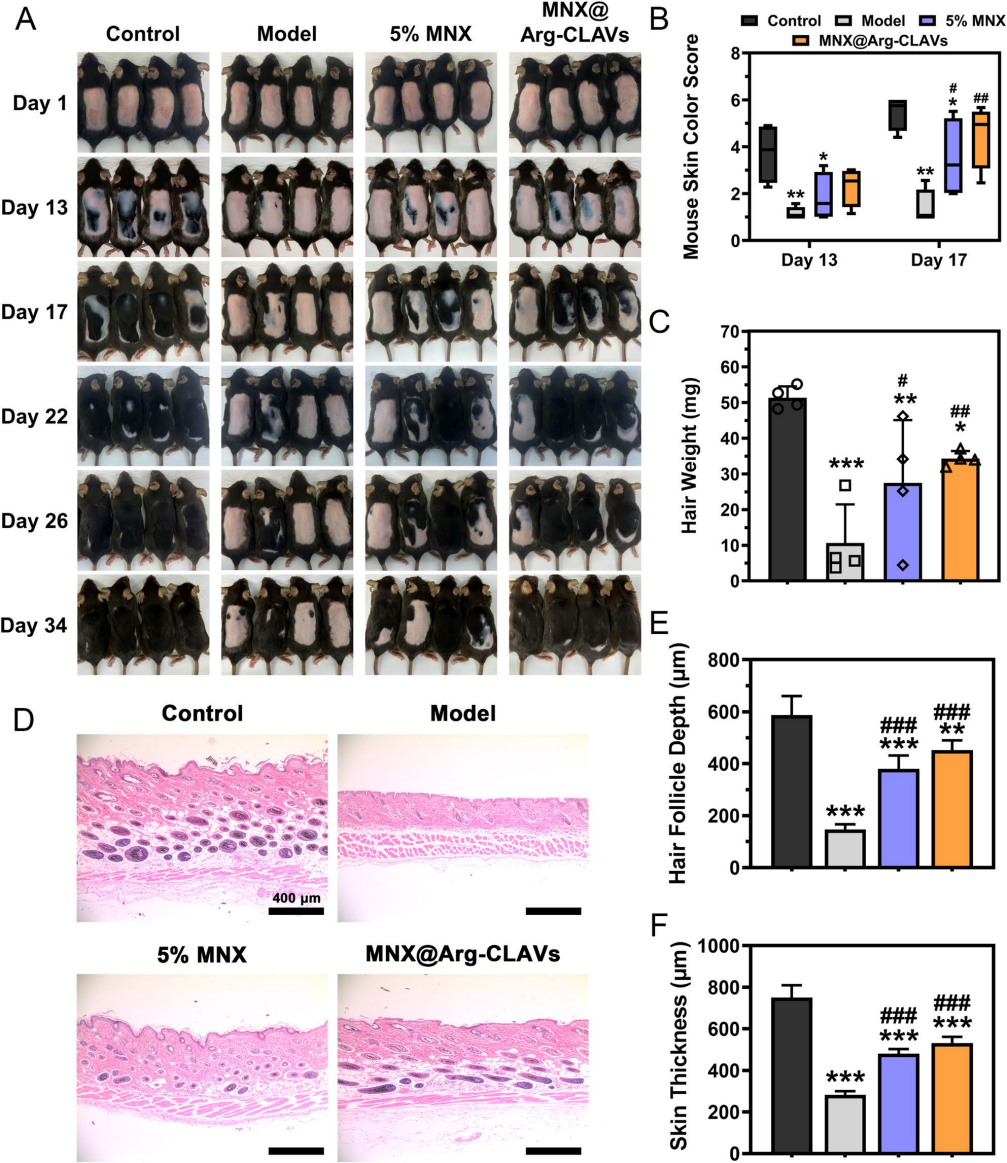
In vivo efficacy evaluation of nanovesicles on AGA female model. The photo of hair regeneration (**A**), skin color score (**B**), and the weight of regenerated hair (**C**). The H&E staining of vertical skin (**D**) (bar = 400 μm), hair follicle depth (**E**), and skin thickness (**F**). ^*^P ˂ 0.05, ^**^P ˂ 0.01, ^***^P ˂ 0.001 vs. Control. ^#^*P* ˂ 0.05, ^##^*P* ˂ 0.01, ^###^*P* ˂ 0.001 vs. Model

In the female AGA model, distinct pathological characteristics were observed: compared to the control group, the model group showed a significantly prolonged time for skin darkening (Fig. 12A). Over half of the mice remained hairless at experimental termination, thereby validating successful TS-induced AGA induction in female mice. By day 17, both 5% MNX and MNX@Arg-CLAVs groups demonstrated early changes in skin pigmentation, with significantly elevated skin color scores compared to the model group (Fig. 12B). On day 34, MNX@Arg-CLAVs-treated mice achieved complete hair regrowth, whereas the 5% MNX group exhibited marked individual variation, with one in four still largely bald. Meanwhile, the new hair weight of MNX@Arg-CLAVs group, reaching 34.35 ± 1.21 mg, also outperformed both 5% MNX (27.52 ± 1.45 mg) and the model group (10.67 ± 1.89 mg) (Fig. 12C).

Histopathological evaluation of female AGA mice was presented in Fig. 12D. Compared to controls, the model group exhibited reduced density of hair follicles, shallow follicular implantation depth (Fig. 12E), and decreased skin thickness (Fig. 12F). Treatments with 5% MNX and MNX@Arg-CLAVs promoted deep dermal migration of DP, inducing new hair formation while increasing skin thickness to support the development of HFs. Notably, MNX@Arg-CLAVs demonstrated marginally superior efficacy compared to the commercial MNX tincture, suggesting enhanced therapeutic potential through optimized nanocarrier delivery. Taken together, MNX@Arg-CLAVs also exerted outstanding hair-promotion activity in female AGA mice, showing great therapeutic potential in both male and female AGA treatment.

### In vivo biosafety

The in vivo safety of nanovesicles was evaluated by recording changes in body weight and detecting organ lesions. During the experimental period, there were no significant changes in body weight observed among different treatment groups, maintaining relatively stable levels (Figure S9A). As demonstrated in Fig. 9C, prolonged administration of the drug formulation did not induce visible erythema or edema on the skin surface of treated mice, suggesting low cutaneous irritability. Histopathological analysis of major organs (heart, liver, spleen, lung, and kidney) revealed no significant morphological alterations compared with control groups. Specifically, H&E staining showed normal tissue architecture with regular cellular arrangement and absence of inflammatory cell infiltration in all examined organs (Figure S9B). In brief, these findings indicated that both Arg-CLAVs and MNX@Arg-CLAVs exhibited satisfactory in vivo safety profiles, thereby supporting their long-term application capacity in AGA therapy.

## Discussion

AGA pathophysiology critically involves an interplay between the genetic makeup of affected individuals and androgens, which detrimentally affect hair growth-promoting signaling factor levels by HF cells. Extensive research has been conducted to enhance the hair-inducing capacity of DPCs, and remodel impaired HF niche, aiming to initiate HF regeneration [31].

PUFAs can mainly be classified into *ω*−3 type and *ω*−6 type based on the position of their double bonds. PUFAs also play a crucial role in the organogenesis and functional maintenance of the skin. A deficiency of PUFAs can lead to skin lesions [9], such as scalp dermatitis, hair loss and hair discoloration [32]. Ryu et al. discovered that the linoleic acid present in the seeds of wild mustard can upregulate the expression of various growth factors and promote the proliferation of DPCs [33]. Zeng et al. discovered that various PUFAs could treat hair loss by exerting antioxidant effects and inhibiting ferroptosis in DPCs [34]. In our study, we identified that CLA not only promoted HDPCs proliferation but relieved the DHT-induced cell damage. For better HF-targeting effect and skin feel, we developed a nanovesicle based on CLA (Arg-CLAVs) that serves as a versatile platform for synergizing with existing drugs. Through in vitro pharmacological tests, we found that, compared to free CLA, Arg-CLAVs exhibited significantly enhanced activity in remodeling the HF niche by stimulating neovascularization, scavenging oxidative stress, and enhancing proliferation and migration of HDPCs.

However, recent clinical analysis has revealed that DHT drives HDPCs aging. Biopsy of AGA bald scalps revealed significant DNA damage in DPCs along with upregulated expression of the senescence marker *CDKN2A* (*p16INK4a*), indicating a strong pathological correlation between AGA and cell aging [35]. Additional in vitro studies have shown that exposure to high-level DHT impairs cell vitality, enhances β-galactosidase staining density, arrests cell cycle and elevates intracellular ROS levels in DPCs [36]. These aging-triggered chain events, if not intervened timely, may even lead to permanent loss of HF function. The current therapies primarily focus on modulating DHT levels and enhancing angiogenesis, yet largely neglect the critical role of HFs aging. That would bring about dissatisfied outcomes. In our study, we found that low-dose DHT could induce an increase in the staining rate of β-galactosidase in HDPCs and an elevation in ROS, while high-dose DHT leads to cell damage and death. When counteracting the effect of DHT, combating cellular damage and delaying aging is equally crucial.

In vitro, by measuring cell viability, β-galactosidase staining intensity, ROS level, SASP level (e.g. TNF-α, IL-6, TGF-β1), Arg-CLAVs remarkably inhibited DHT-induced cell senescence. We also analyzed the expression levels of molecules associated with cell senescence and hair-inducing capacity of HDPCs. Gene expression levels of *SRD5A2*, *CDKN2A*, and *p53* were downregulated, while *β-Catenin*, *FGF-7*, and *IGF-1* were upregulated in the Arg-CLAVs group. We hypothesized that Arg-CLAVs alleviate HF aging by modulating HDPCs pathways. To elucidate this mechanism, we explore RNA-seq combined with KEGG & GO enrichment analyses. These analyses have localized the impact of Arg-CLAVs on DHT-treated HDPCs to the MARK-ERK pathway. The result of western blotting further confirmed the analyses, characterized by upregulated phosphorylation of the ERK level treated after Arg-CLAVs. P-ERK through an MAPK–ERK cascade is a major effect in skin epithelium [37], including protecting HFSC from inflammatory stress [38]. These findings collectively suggest that Arg-CLAVs can fight against HF senescence by triggering the phosphorylation of the MAPK-ERK pathway.

MNX is the only FDA-approved topical formulation for AGA treatment, which is recognized to improve alopecia through vasodilatation. We hypothesized that Arg-CLAVs can enhance the hair growth effect of MNX as a booster, and make up for the deficiency of MNX in anti-cell aging. In the male AGA model, MNX@Arg-CLAVs, only containing 0.45% MNX, significantly outperformed equivalent-dose MNX tinctures and even commercial 5% MNX tinctures. Besides, MNX@Arg-CLAVs has improved the individual differences of MNX. In fact, some studies have shown that the sulfation of MNX catalyzed by sulfotransferases (*SULT1A1*) is a critical step in the hair-growth effects of MNX, and the activity of this enzyme displays individual variability [30]. However, TPGS in the lipid layer of MNX@Arg-CLAVs could enhance the activity of sulfotransferases as reported already, which might be the reason for the reduction of individual differences. Consistent with these observations, tissue immuno-histochemical staining showed that the staining intensity of *p53* (a marker of cell aging) was significantly decreased in the dermal layer and HFs. Ki67 (a marker of cellular proliferation) and CK19 (a marker of HFSC activation) displayed higher fluorescence intensity in the MNX@Arg-CLAVs group. The results in the female AGA model were aligned with the male AGA model. These findings support our hypothesis that Arg-CLAVs compensate for the anti-aging activity of MNX. Our study demonstrates that Arg-CLAVs can relieve HF aging and promote HF growth in AGA. Besides, Arg-CLAVs have a simpler preparation method, and the content of organic solvents is lower, making it suitable for production and clinical use. However, due to the limited sample size and experiment conditions, the efficacy and safety of Arg-CLAVs need more careful investigation. Moreover, how to promote the growth of physiological aging-related HF, requires further research. In future studies, we will conduct more in-depth and exploratory studies of these aspects to achieve superior HF regrowth.

## Conclusion

In conclusion, this study presents a multifunctional nanoplatform Arg-CLAVs not only counteract androgen-induced follicular senescence and restore the HF niche to drive hair regeneration but also serve as a carrier to enhance the efficacy and safety of existing drugs (e.g., MNX) by reducing dosage and adverse effects. Specifically, Arg-CLAVs protected HDPCs from DHT-induced cell senescence by inhibiting HDPCs injury, reducing β-galactosidase staining intensity, eliminating intracellular ROS, and suppressing the secretion of SASP components. It also promoted vascularization of HUVECs, alleviated the oxidative stress, and enhanced the proliferation and migration of HDPCs and HUVECs, collectively restoring a pro-regenerative HFs niche. MNX@Arg-CLAVs loading 0.45%MNX achieved comparable hair growth-promoting efficacy to commercial 5% MNX tincture in both male and female AGA models, while significantly reducing organic solvent content and minimizing skin irritation. In vivo studies further demonstrated that MNX@Arg-CLAVs mitigated aging-induced collagen fiber degradation and decreased *p53* expression compared to the commercial MNX tincture. In addition, our findings revealed that Arg-CLAVs reactivated that DHT-suppressed MAPK-ERK pathway. This study provides an innovative therapeutic strategy for AGA and offers new insights into the development of nanocarrier-based interventions for hair loss disorders.

## Supplementary Information

Supplementary Material 1.

## Data Availability

No datasets were generated or analysed during the current study.
