# Supplementary material for: Rejuvenating senescent hair follicles: a novel conjugated linoleic acid based nanovesicle approach to treat androgenic alopecia
Source: J Nanobiotechnology. 2025 Nov 26;23:754. doi: 10.1186/s12951-025-03865-2 (PMC12673742; doi:10.1186/s12951-025-03865-2)
Supplement: Supplementary file 1 — Supplementary Material 1. [file 12951_2025_3865_MOESM1_ESM.docx]

**Supplementary Materials**

Rejuvenating Senescent Hair Follicles: A Novel Conjugated Linoleic Acid Based Nanovesicle Approach to Treat Androgenic Alopecia

Yating Dong *^a,^* ^*^, Yingying Sun *^a, *^*, Aojie Li *^a^*, Yuxuan Yu *^a^*, Wengkuan U *^a^*, Hongjuan Zhang *^a^*, Xuefei Zhang *^b^*, Yihua Huang *^a, #^*, Haiyan Hu *^a, c, d, #^*

*^a^ School of Pharmaceutical Sciences, Sun Yat-sen University, University Town, Guangzhou 510006, P. R. China*

*^b^ School of Traditional Dai-Thai Medicine, West Yunnan University of Applied Sciences, Jinghong 666100, P. R. China*

*^c^* *State Key Laboratory of Anti-Infective Drug Discovery and Development, School of Pharmaceutical Sciences, Sun Yat-sen University, Guangzhou 510006, P. R. China*

*^d^ Guangdong Provincial Key Laboratory of Chiral Molecule and Drug Discovery, Sun Yat-sen University, University Town, Guangzhou 510006, P. R. China*

^*^ These authors contributed equally to this work.

^#^ Correspondence: Yihua Huang, email: [huangyh376@mail2.sysu.edu.cn](mailto:huangyh376@mail2.sysu.edu.cn).

Prof. Haiyan Hu, email: lsshhy@mail.sysu.edu.cn.

**Table S1** The primer sequence for RT-qPCR

| Gene | Primer sequence |
| --- | --- |
| *GADPH* | F: GGAGCGAGATCCCTCCAAAAT |
|  | R: GGCTGTTGTCATACTTCTCATGG |
| *β-Catenin* | F: CATCTACACAGTTTGATGCTGCT |
|  | R: GCAGTTTTGTCAGTTCAGGGA |
| *FGF-7* | F: TTGTGGCAATCAAAGGGGTG |
|  | R: CCTCCGTTGTGTGTCCATTTAGC |
| *IGF-1* | F: GCTCTTCAGTTCGTGTGTGGA |
|  | R: GCCTCCTTAGATCACAGCTCC |
| *SRD5A2* | F: CGGTTTAGCTTGGGTGTCTTC |
|  | R: CCGAGGAAATTGGCTCCAGAA |
| *CDKN2A* | F: GGGTTTTCGTGGTTCACATCC |
|  | R: CTAGACGCTGGCTCCTCAGTA |
| *p53* | F: CAGCACATGACGGAGGTTGT |
|  | R: TCATCCAAATACTCCACACGC |


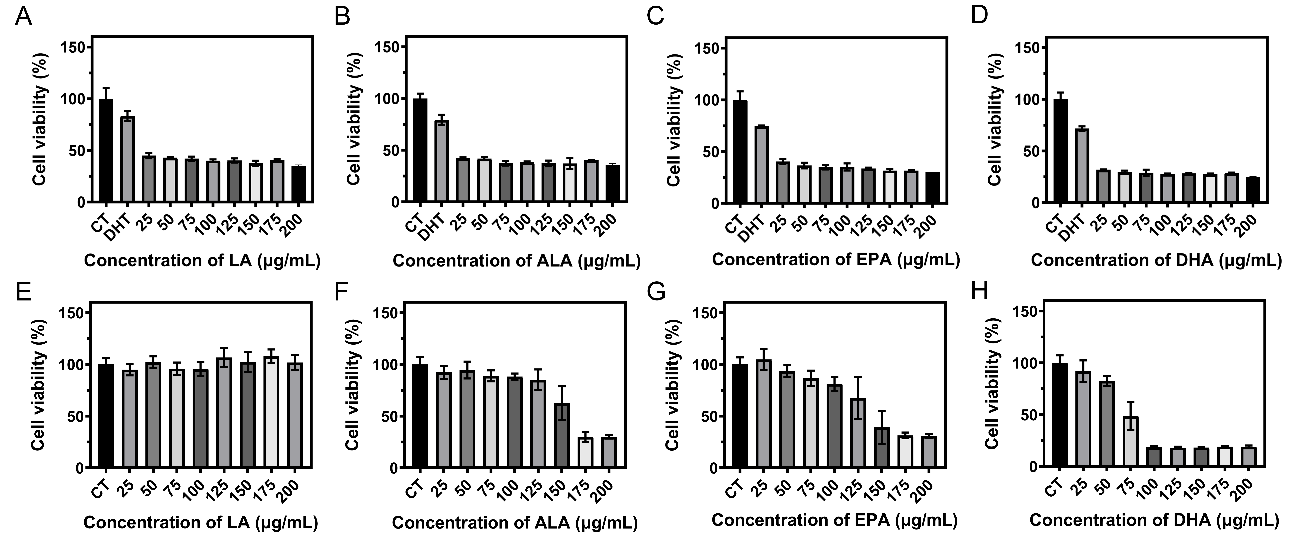


**Figure S1** The effect on protecting HDPCs from DHT damage of LA (A), ALA(B), EPA (C), and DHA (D), and the effect on promoting HDPCs proliferation of LA (E), ALA(F), EPA (G), and DHA (H).


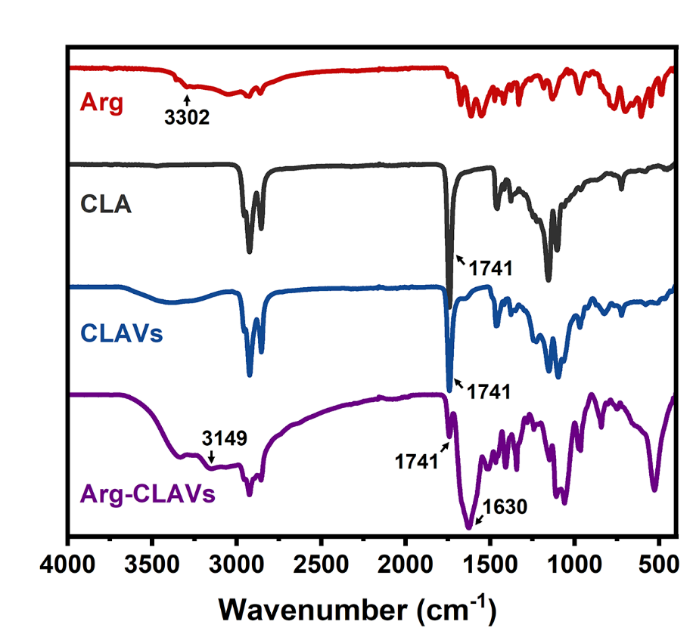


**Figure S2** The FITR spectrum of Arg, CLA, CLAVs and Arg-CLAVs.


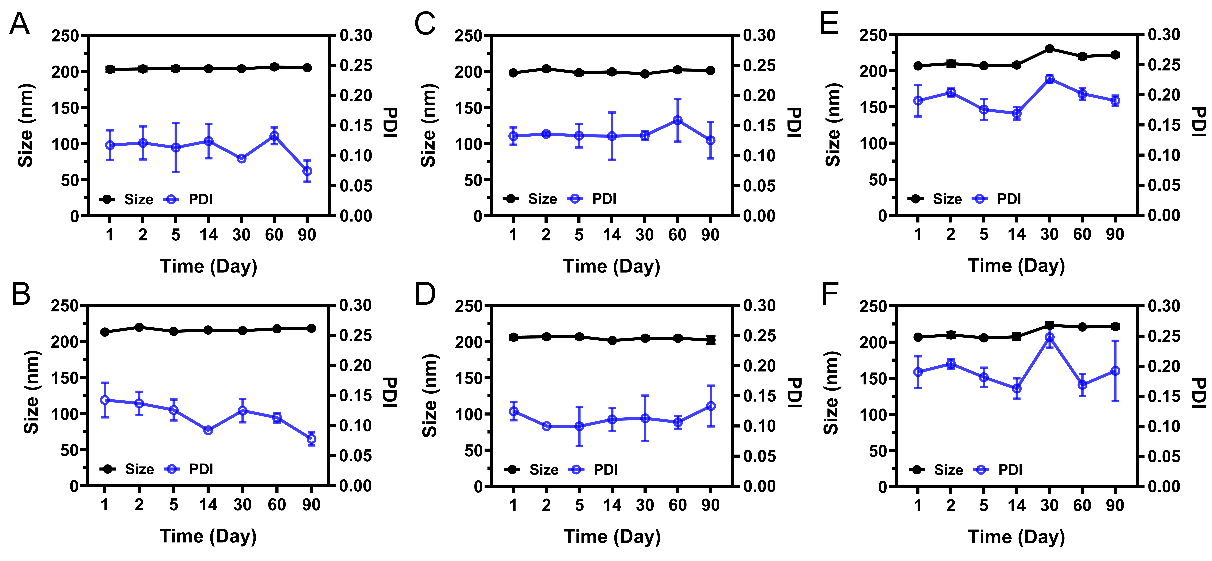


**Figure S3** The stability of CLAVs at RT (A) and 4 ℃ (B), Arg-CLAVs at RT (C) and 4 ℃ (D), and MNX@Arg-CLAVs at RT (E) and 4 ℃ (F).

**
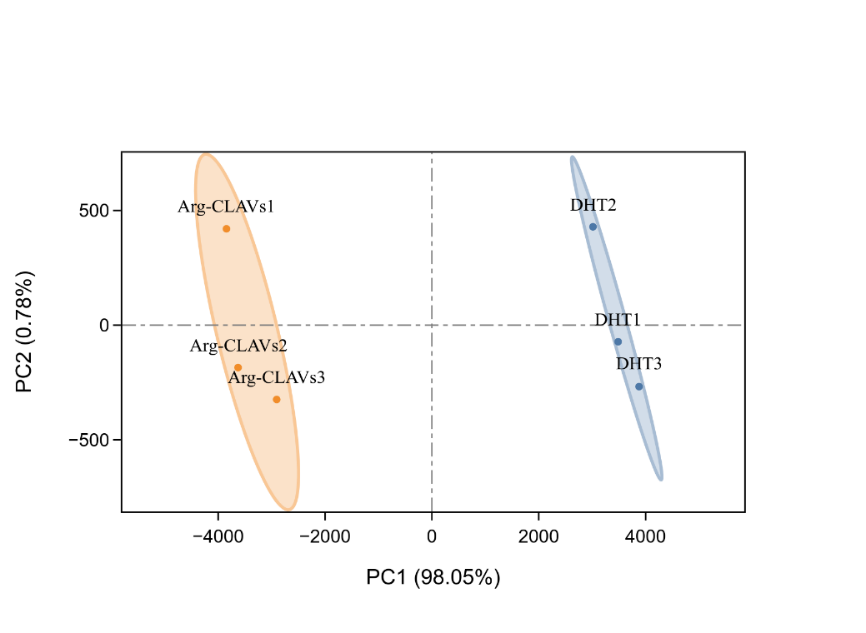
**

**Figure S4** The principal component analysis of HDPCs after DHT (blue) and Arg-CLAVs (orange) treatments (n=3).


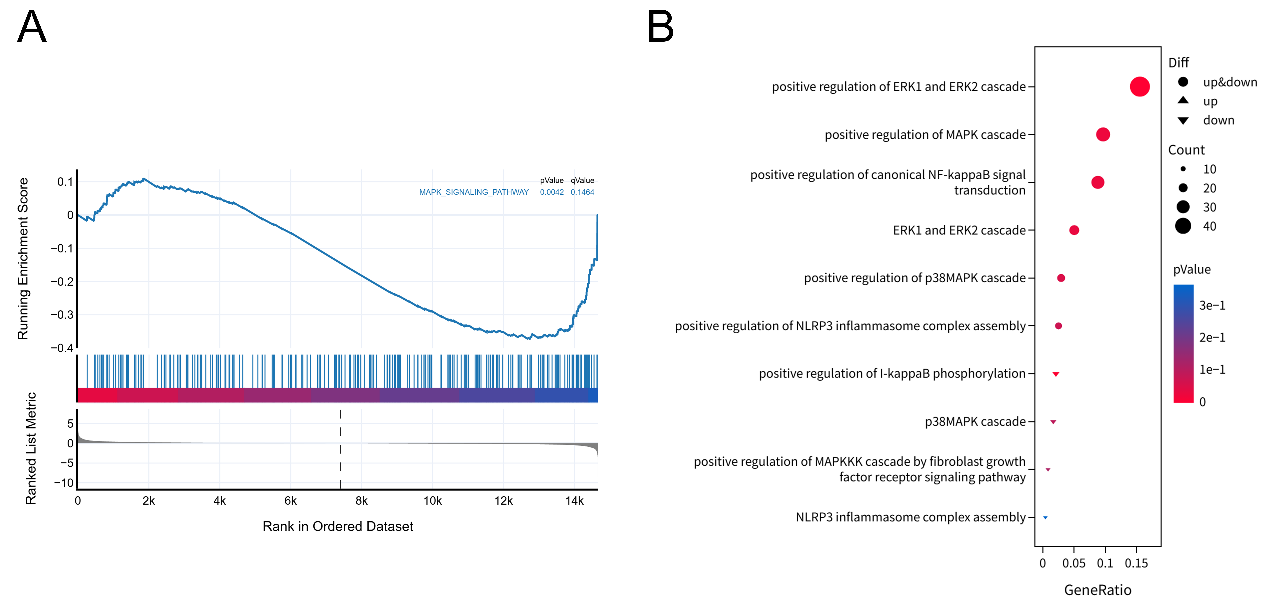


**Figure S5** The GSEA (A) and GO enrichment analysis (B) and of MAPK-ERK pathway. FDR < 0.25.


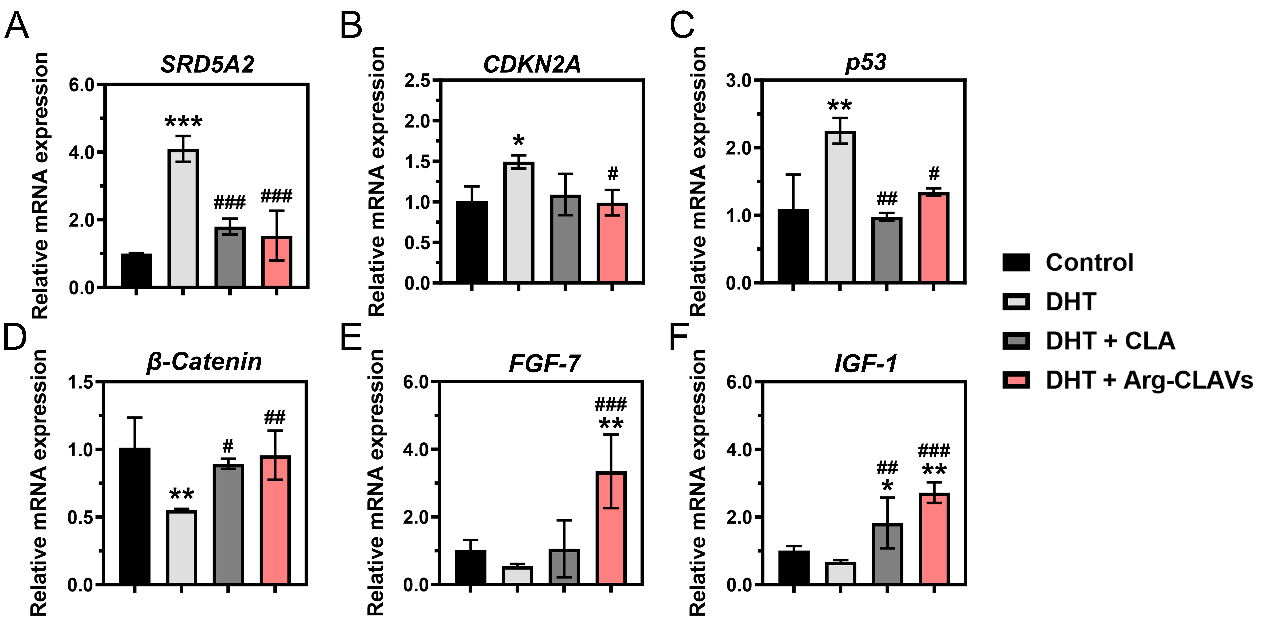


**Figure S6** The mRNA expression in HDPCs detected by RT-qPCR: The mRNA expression levels of *SRD5A2* (A), *CDKN2A* (B), *p53* (C), *β-Catenin* (D), *FGF-7* (E), and *IGF-1* (F). ^*^*P* < 0.05, ^**^*P* < 0.01, ^***^*P* < 0.001 *vs.* Control. ^#^*P* < 0.05, ^##^*P* < 0.01, ^###^*P* < 0.001 *vs.* DHT.

**Table S2** The EE and DL of MNX@Arg-CLAVs

| Formulation | EE% | DL% |
| --- | --- | --- |
| MNX@Arg-CLAVs | 97.14 ± 1.89% | 4.86 ± 0.13% |


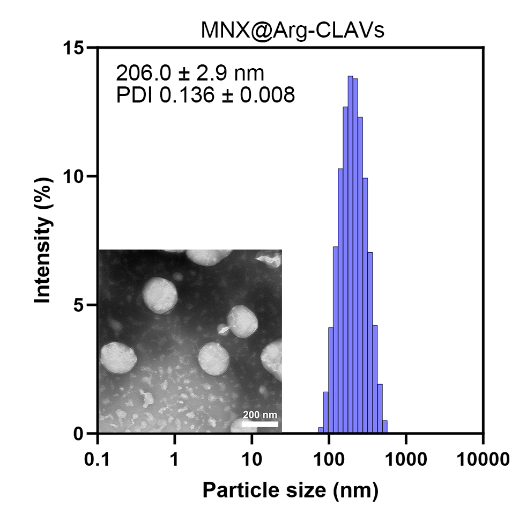


**Figure S7** The particles size, size distribution, PDI, and TEM image of MNX@Arg-CLAVs.

**Table S3** The differences between commercial MNX tincture and MNX@Arg-CLAVs

| Formulations | MNX content (mg/mL) | Organic solvents content (%) | Propylene glycol content (%) |
| --- | --- | --- | --- |
| Commercial MNX tincture | 50 | 75 ~ 85 | 50 |
| MNX@Arg-CLAVs | 4.5 | 10 | nil |


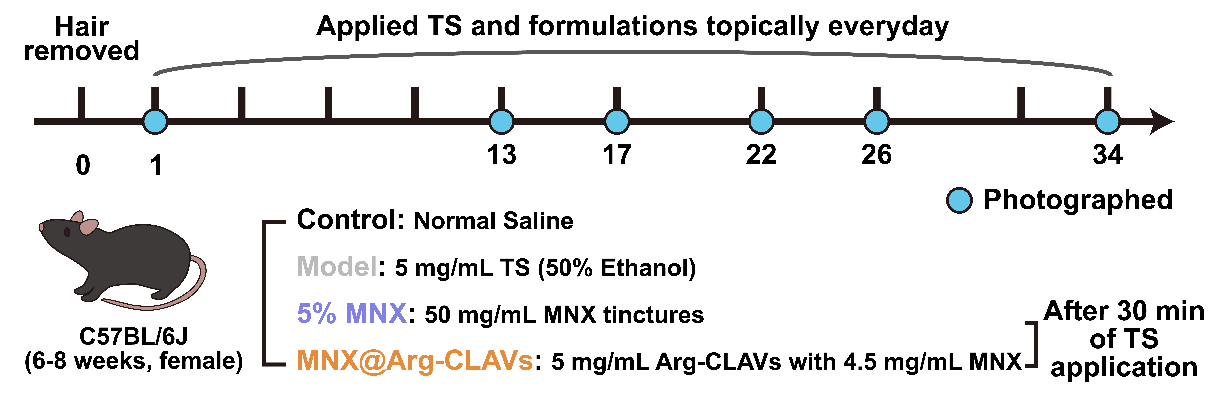


**Figure S8** The establishment of AGA female model, and the treatment schedule.


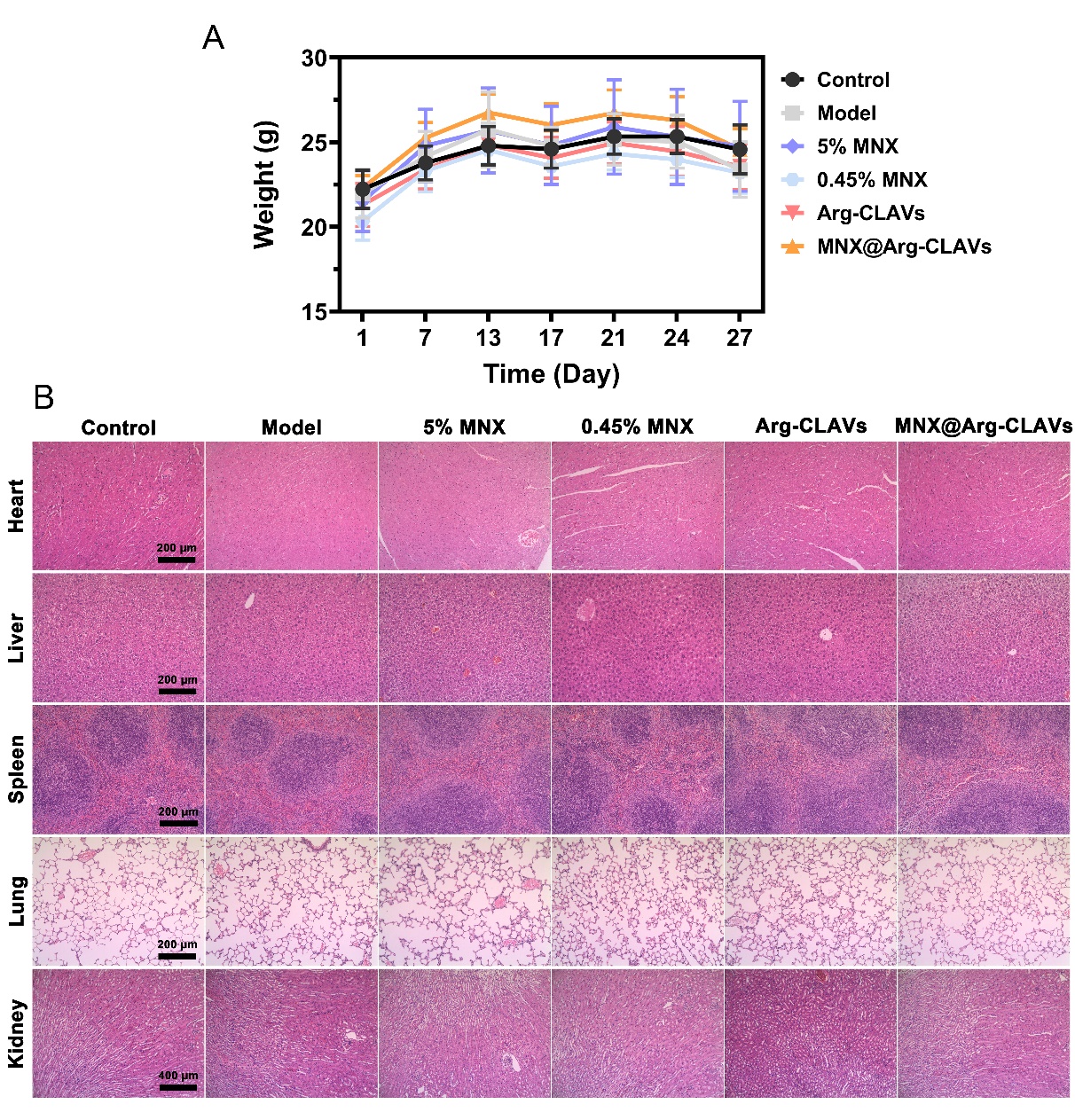


**Figure S9** The weight changes (A) and the H&E staining of organs (B) in male AGA mice after being treated by different formulations. Bar = 200 μm
